# Neutrophil-Mediated Immunopathology and Matrix Metalloproteinases in Central Nervous System – Tuberculosis

**DOI:** 10.3389/fimmu.2021.788976

**Published:** 2022-01-12

**Authors:** Xuan Ying Poh, Fei Kean Loh, Jon S. Friedland, Catherine W. M. Ong

**Affiliations:** ^1^ Infectious Diseases Translational Research Programme, Department of Medicine, Yong Loo Lin School of Medicine, National University of Singapore, Singapore, Singapore; ^2^ Institute for Infection and Immunity, St George’s, University of London, London, United Kingdom; ^3^ Division of Infectious Diseases, Department of Medicine, National University Hospital, Singapore, Singapore; ^4^ Institute for Health Innovation and Technology (iHealthtech), National University of Singapore, Singapore, Singapore

**Keywords:** tuberculosis, central nervous system tuberculosis, neutrophils, matrix metalloproteinases, stroke, host-directed therapy

## Abstract

Tuberculosis (TB) remains one of the leading infectious killers in the world, infecting approximately a quarter of the world’s population with the causative organism *Mycobacterium tuberculosis* (*M. tb*). Central nervous system tuberculosis (CNS-TB) is the most severe form of TB, with high mortality and residual neurological sequelae even with effective TB treatment. In CNS-TB, recruited neutrophils infiltrate into the brain to carry out its antimicrobial functions of degranulation, phagocytosis and NETosis. However, neutrophils also mediate inflammation, tissue destruction and immunopathology in the CNS. Neutrophils release key mediators including matrix metalloproteinase (MMPs) which degrade brain extracellular matrix (ECM), tumor necrosis factor (TNF)-α which may drive inflammation, reactive oxygen species (ROS) that drive cellular necrosis and neutrophil extracellular traps (NETs), interacting with platelets to form thrombi that may lead to ischemic stroke. Host-directed therapies (HDTs) targeting these key mediators are potentially exciting, but currently remain of unproven effectiveness. This article reviews the key role of neutrophils and neutrophil-derived mediators in driving CNS-TB immunopathology.

## Introduction

The global tuberculosis (TB) incidence remains in epidemic proportions, with an estimated 10 million new TB cases and 1.4 million TB deaths in 2019 (1). Central nervous system tuberculosis (CNS-TB), which accounts for a minimum 1-2% of all TB disease and 5-10% of all extra-pulmonary TB disease, is the most devastating manifestation of TB with high mortality and neurological morbidity (2). CNS-TB is almost certainly under-diagnosed and under-reported. CNS-TB encompasses three clinical-pathological forms: tuberculous meningitis (TBM), tuberculomas, and tuberculous brain abscess (3). TBM is the most severe manifestation of CNS-TB, with most untreated TBM patients dying within 5-8 weeks of disease onset (4). The pathogenesis of CNS-TB, is believed to originate from the lung, where *M. tb* primarily infects, followed by lympho-hematogenous dissemination and crossing the blood-brain barrier (BBB) to the brain, causing CNS-TB (5). During *M. tb* infection, a complex interplay between host immune cells and *M. tb* virulence factors determines whether the mycobacteria can be contained or progress to clinical TB disease. However, the underlying mechanisms of CNS-TB immunopathology are not fully understood.

Neutrophils are increasingly recognized as key mediators of TB immunopathology. Necrotizing granulomas containing neutrophils and neutrophil influx at the site of infection are hallmarks of active TB disease in humans (6–8). The presence of neutrophils in human CNS tuberculomas further highlight their role in this disease (9). There is accumulating evidence that neutrophil-derived mediators including matrix metalloproteinase-9 (MMP-9) and tumor necrosis factor-α (TNF-α) result in immunopathology in CNS-TB (10–13). Neutrophils may also crosstalk with other immune cells such as macrophages to upregulate cytokine secretion (14), while their interactions with activated platelets may result in thrombosis, leading to the occurrence of ischemic stroke (15, 16). In this review, we discuss the diverse roles of neutrophils in driving CNS-TB immunopathology, highlighting key neutrophil mediators including matrix metalloproteinases. We summarize the current research on adjunctive therapies in CNS-TB, such as steroids, aspirin and anti-TNF-α and discuss future potential therapies to improve outcomes of this disease.

## Epidemiology of CNS-TB

The incidence of extra-pulmonary TB has increased in recent years since the onset of human immunodeficiency virus (HIV) infection and increased immigration from TB endemic regions (17). The exact global incidence of CNS-TB is unknown, given the diagnostic challenges and lack of microbiological confirmation in many suspected CNS-TB cases, which results in under-reporting (18). Regional studies have documented prevalence rates of TBM between 0.9-2.2% of all TB cases (19–22), and affluent countries like Canada and the United States of America are similarly afflicted (23, 24). The risk factors for CNS-TB include young age (25) and immunocompromised individuals such as patients living with HIV/AIDS (PLHA) (26–28). PLHA are five times more likely than HIV-negative individuals to develop neurological manifestations of TB, with up to 40% of CNS-TB-HIV co-infected patients succumbing while on anti-retroviral therapy (18, 29–31). Not only are children at greater risk of developing CNS-TB than adults, they are also significantly more likely than adults to suffer long-term neurological sequelae (32, 33). While younger age and HIV co-infection were associated with microbiologically proven CNS-TB, older age was associated with increased mortality (34). Diabetes mellitus, chronic kidney failure, presence of hydrocephalus and microbiologically-confirmed CNS-TB were independent risk factors for increased mortality (19, 32, 34–37).

## Pathogenesis and Pathology of CNS-TB

In the lung microenvironment, infection of alveolar macrophages by *M. tb* activates an innate inflammatory immune response rapidly (38) followed by a predominantly T-helper 1 (Th1) immune response which eventually leads to granuloma formation (39). Early in this process before the infection is contained, *M. tb* filter into draining lymph nodes, and most likely enter the circulatory system through the thoracic duct into the subclavian vein (40). A low level of *M. tb* may subsequently disseminate to distant organs such as the brain. Once *M. tb* crosses the BBB and enters the immune-privileged CNS, the limited local innate immune response may facilitate survival and replication of the pathogen (41). A complex interplay of host immune factors and *M. tb* virulence factors determines if the infection is successfully contained or to what extent the infection progresses to clinical TB disease.

The understanding of TBM pathogenesis originated from guinea pig and rabbit studies conducted by Rich and McCordock in 1933 (42). These authors first demonstrated that the meninges could not be directly infected by the hematogenous spread of *M. tb* in these animals, but rather required the direct inoculation of *M. tb* into the CNS to produce TBM (42). From human post-mortem examinations, Rich and McCordock showed that in almost every TBM patient, there was a meningeal focus from which *M. tb* could enter the subarachnoid space and cause meningitis (42). Subsequent studies corroborated their findings, and it became accepted that a caseating vascular focus, termed the “Rich focus”, located in the meninges or adjacent to the ventricles is the key pathway for *M. tb* to gain access to the subarachnoid space and cause a granulomatous infection of the meninges (43–46). The location of these foci and the ability or inability of the host immune response to control the infection determines the form of CNS-TB that develops (5). When a Rich focus in the meninges ruptures, *M. tb* is released from the granulomatous lesions into the CSF, resulting in extensive inflammation and TBM (39). Separately, the enlargement of Rich foci in the brain parenchyma without rupturing gives rise to tuberculomas, thus they often occur in the absence of TBM (39). While it is widely accepted that the rupture of Rich foci causes *M. tb* dissemination leading to TBM, the foci may not present in all CNS-TB manifestations. In rare cases, *M. tb* infection spreads to the CNS from a site of tuberculous otitis or calvarial osteitis (3). Furthermore, histological examination of TBM brain specimens showed that most of the intraparenchymal granulomas are an extension of leptomeningeal lesions, which opposes the Rich focus hypothesis (47). We suspect that alternative *M. tb* entry routes exist to spread to the CNS may not be well characterized.

CNS-TB presents as several forms of intracranial and spinal TB manifestations, with the most common being TBM, tuberculomas, and tuberculous brain abscess. TBM is the most severe manifestation of CNS-TB with highest mortality and neurological morbidity (48, 49). Majority of TBM patients experience non-specific symptoms such as fatigue, fever, malaise, anorexia, and myalgia for 2-8 weeks before the meningitic state ensues, where patients present with headache, fever, vomiting, photophobia and neck stiffness in 75% of cases (50–52). Left untreated, most TBM patients die within 5-8 weeks of disease onset (4). Common radiological features seen in TBM include basal meningeal enhancement, tuberculomas, hydrocephalus, and infarctions (29, 53). Although a combination of these imaging features is highly specific for TBM (95-100%), most radiological findings by themselves lack adequate sensitivity as they may not be detected radiographically until advanced stages (18, 54–56). Hydrocephalus, which is the most frequent cause of raised intracranial pressure in TBM patients, was also found to be associated with advanced stage of infection, with high morbidity and mortality (18, 57). Cerebral vasculitis and inflammation both of which are regulated by platelet activation result in infarcts and are the primary cause of permanent brain tissue damage in TBM (58–60).

Cranial nerve palsies occur in 25-50% of patients and can lead to vision loss if the optic nerve is involved (2). Although seizures occur in 10-15% of patients, it is more common in pediatric TBM patients (18). The severity of TBM disease can be classified into 3 grades based on modifications of the Medical Research Council staging system (**Table 1**) (65), which has been shown by numerous reports to have considerable prognostic value (61, 62, 66). In HIV-negative TBM patients, mortality has been documented to be 20% at stage I, 30% at stage II, and 55% at stage III (62).

**Table 1:** British medical research council clinical criteria for staging TBM.

Stage/grade	Classic criterion	Contemporary criterion
**I**	Fully conscious and no focal deficits	Alert and oriented without focal neurological deficits
**II**	Conscious but with inattention, confusion, lethargy, and focal neurological signs	Glasgow coma score of 14-11 or 15 with focal neurological deficits
**III**	Stuporous or comatose, multiple cranial nerve palsies, or complete hemiparesis or paralysis	Glasgow coma score of 10 or less, with or without focal neurological deficits

Adapted from (61–64).

The tuberculoma is the pathological hallmark of *M. tb* infection, and may occur with or without TBM development (39). Macroscopically, they appear as spherical, encapsulated space-occupying lesions on neuroradiology (67). While a solitary lesion is more common in CNS-TB patients, multiple tuberculomas or even up to >100 tuberculomas have been seen in exceptional cases (68). Most tuberculomas are up to 1 cm in diameter, with approximately 10% between 1-3 cm, but may reach sizes of up to 8 cm (69). Microscopically, a tuberculoma is characterized by a granulomatous region, comprising of epithelioid cells, Langerhans giant cells and lymphocytes, and often a central area of caseating necrosis (29).

Tuberculous brain abscess is a rare manifestation of CNS-TB. Its appearance is more similar to pyogenic brain abscess than to tuberculomas and generally larger in size than tuberculomas. These brain abscesses may be unilocular or multilocular, and is characterized by cavity formation with central area of pus containing viable bacteria (70, 71).

Analysis of the pathology in TBM patients reveal three types of granulomas found mainly in the leptomeninges. Non-necrotizing granuloma comprises of activated macrophages, lymphocytes and plasma cells, while necrotizing gummatous granuloma containing reticulin fibers are present in the necrosis central area with intact neutrophils (47). The other necrotizing abscess-type of granuloma has similar presentation to tuberculous brain abscess, which consists of pus with high concentration of neutrophils (71, 72). The abundance of neutrophils indicates their important role in CNS-TB.

## Cellular Responses in CNS-TB

**Figure 1:**
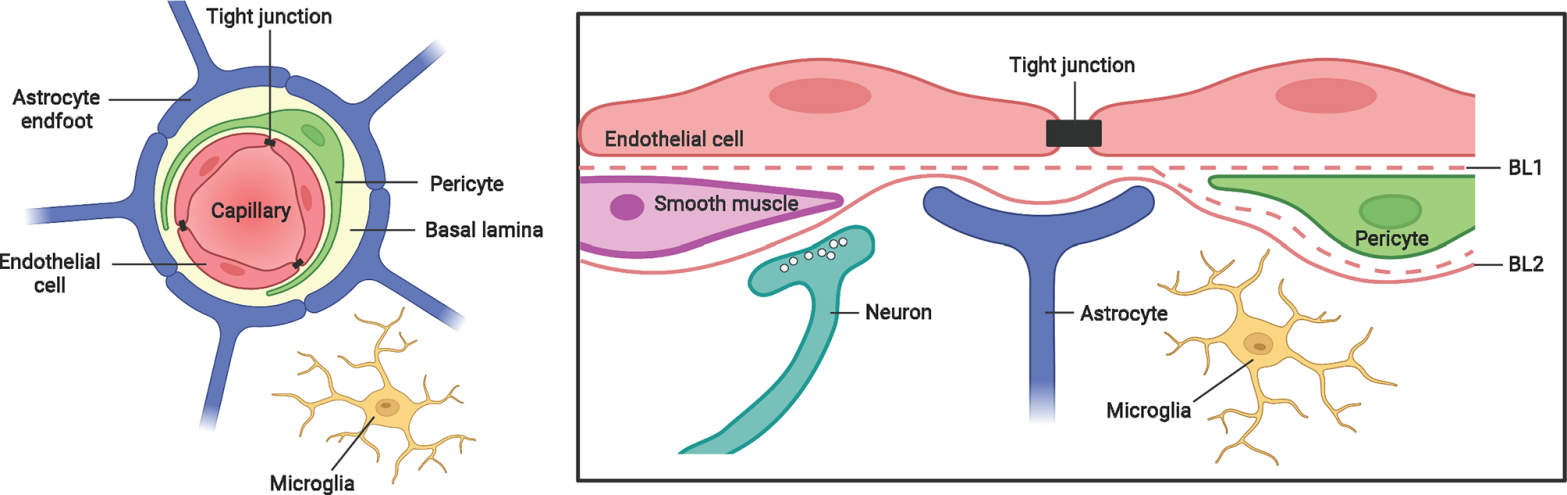
The blood-brain-barrier structure and cellular composition. The BBB is a highly complex structure, made up of brain microvascular endothelial cells, pericytes, astrocytes and a non-cellular component – the basal lamina. Tight junctions between brain endothelial cells maintain the integrity and permeability of brain microvessels. Both the endothelial cells and pericytes are enclosed by, and contribute to the perivascular extracellular matrix (basal lamina 1, BL1), which is different in composition from the extracellular matrix of the glial end feet (BL2) bounding the brain parenchyma. Figure created with Biorender.com.

The brain is protected from blood-borne pathogens by the BBB, which consists of brain microvascular endothelial cells joined by tight junctions, astrocyte end-feet ensheathing the capillary, and pericytes embedded in the basement membrane (**Figure 1**) (73). Two mechanisms by which *M. tb* crosses the protective BBB have been proposed (74–76). *In vitro* and animal models have demonstrated that *M. tb* is capable of invading and traversing brain endothelial cells directly by modulating their actin rearrangement (75, 76). The *M. tb* gene *pknD* (*Rv0931c*) was recently identified as a critical virulence factor that facilitates bacterial adhesion to laminin-1 and -2 on brain endothelial cells (77). Another potential route of CNS entry is the “Trojan horse” mechanism, whereby *M. tb* is trafficked across the BBB in infected macrophages and neutrophils (74). Following the breach of the BBB, resident CNS-specific cells such as microglia are activated and leukocytes will infiltrate into the CNS to induce the inflammatory response.

### Crosstalk Between Neutrophils and Other Immune Cells

Leukocytes such as neutrophils and monocytes interact with endothelial adhesion molecules to transmigrate across the BBB. We have shown that conditioned media from monocytes infected with *M. tb* (CoMtb)-stimulation of a BBB cellular model significantly upregulated endothelial adhesion molecules intercellular adhesion molecule 1 (ICAM-1), vascular cell adhesion molecule 1 (VCAM-1), P-Selectin and E-Selectin, which resulted in increased transmigration of monocytes and neutrophils across a BBB model (78).

TNF-α plays an immune-regulatory role in the infiltration of leukocytes in CNS-TB. *M. tb*-infected TNF-knockout (TNF^-/-^) mice showed acute ventriculitis characterized by neutrophil infiltrates extending into the periventricular tissue of the brain, while a mixture of lymphocytes and neutrophils were observed at the choroid plexus (79). Compared to wild type mice, *M. tb*-infected TNF^-/-^ mice showed increased macrophages and dendritic cell (DC) recruitment, with upregulation of chemokines macrophage inflammatory protein-1α (MIP-1α), monocyte chemoattractant protein-1 (MCP-1) and Regulated upon Activation Normal T Cell Expressed and Presumably Secreted (RANTES) (79). In CNS-TB, neutrophils may engage in complex multi-directional interactions with other immune cells such as monocytes, macrophages, dendritic cells and T lymphocytes. Several studies have evaluated the interaction of neutrophils and monocytes in CNS-TB. Monocyte-neutrophil networks resulted in MMP-9 upregulation which may be further upregulated by hypoxia (80), and lead to type IV collagen and tight junction protein (TJP) breakdown with an associated increase in neutrophil and monocyte transmigration across the BBB (78, 79). Using an *in vitro* BBB model, we elucidated the molecular mechanisms by which monocyte-neutrophil networks drive MMP-9 secretion, and found mitogen activated protein kinase (MAPK) and phosphatidylinositide-3 kinase (PI3K)-Akt pathways and the transcription factor nuclear factor kappa B (NF-kB) to regulate neutrophil MMP-9 secretion (9).

As part of the innate host immune response in CNS-TB, neutrophils interact with CNS resident microglia and infiltrated macrophages. In early CNS-TB, both neutrophils and macrophages infiltrate across the BBB into the CNS (78, 79). They are rapidly activated, proliferate and secrete cytokines, which further drive the accumulation and activation of other immune cells (81). *M. tb*-infected neutrophils showed upregulation of MCP-1, which is essential for recruiting macrophages (82, 83). Moreover, Braian et al. demonstrated that macrophages produced significantly higher concentrations of cytokines IL-6, TNF-α, IL-1β and IL-10 in response to NETs from *M. tb*-activated neutrophils but not phorbol myristate acetate-activated neutrophils (14), further highlighting the importance of neutrophil-macrophage interaction in TB infections. Conversely, *M. tb*-infected murine microglia secrete granulocyte-macrophage colony-stimulating factor (GM-CSF) (84), a chemoattractant that may facilitate *M. tb* containment by promoting neutrophil phagocytosis (85).

Neutrophils promote activation of CD4^+^ T cells in TB by facilitating DC migration and antigen presentation (86). Direct *M. tb*-infected DCs showed poor migration, whereas DCs that acquired *M. tb* through uptake of infected neutrophils exhibited unimpaired migration to prime naive CD4^+^ T cells and subsequently activate adaptive immunity (86), implicating the key role of neutrophils in priming the T cells. In CNS-TB, the presence of DCs was reported (79), but no study has investigated whether a similar neutrophil-DC interaction occurs. Further evidence from animal studies demonstrated direct crosstalk between neutrophils and T cells. For instance, *in vivo* depletion of Gr1^+^ neutrophils decreased accumulation of Th1 cells in the lungs of *M. tb*-infected mice (87). Furthermore, Blomgran et al. demonstrated that an inhibition of neutrophil apoptosis in *M. tb*-infected mice delayed the activation of naive CD4^+^ T cells (88). These studies highlight the importance of neutrophils as a bridge between the innate and adaptive immune response.

## Neutrophils in CNS-TB

### Neutrophils Influx

**Figure 2:**
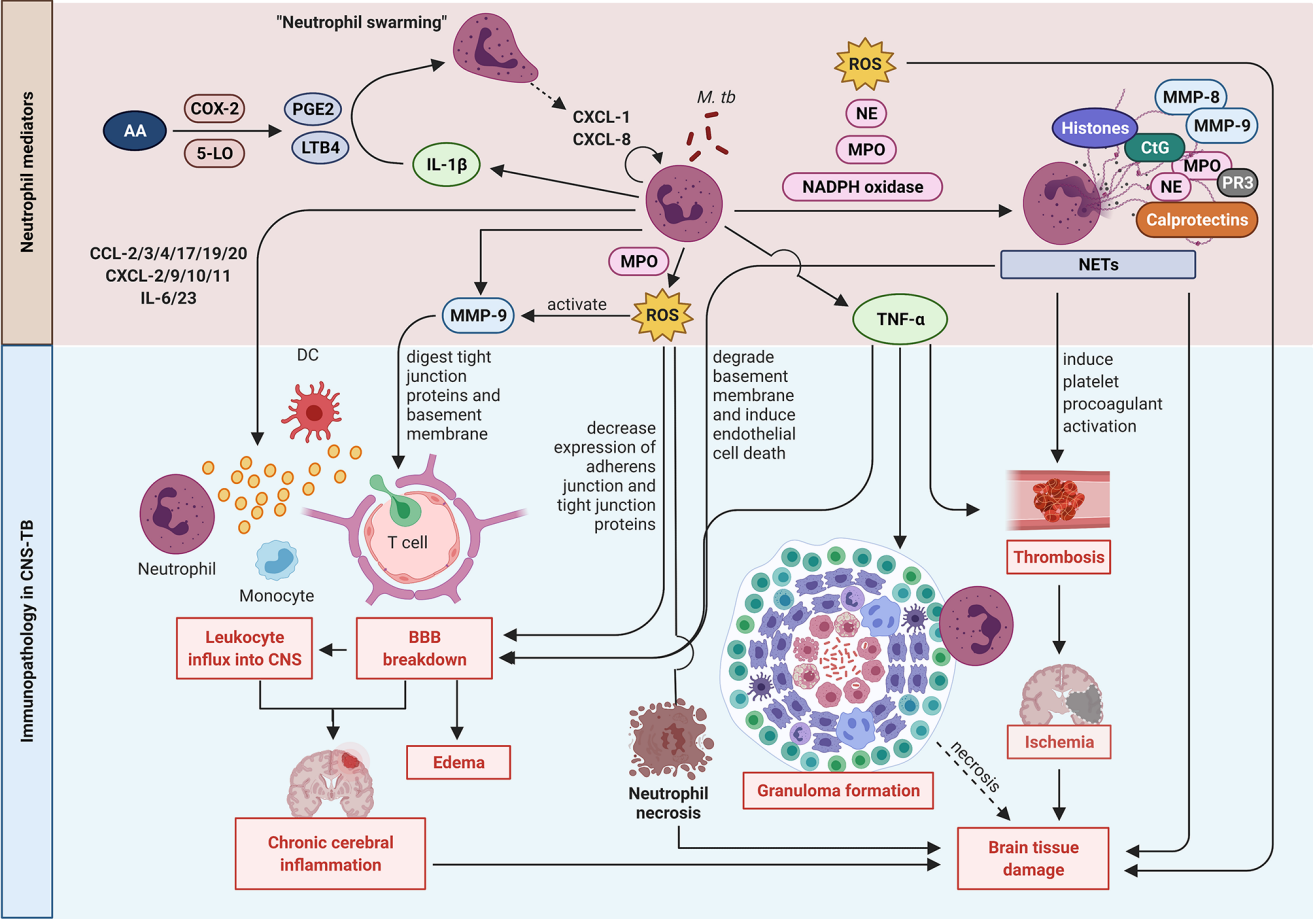
Neutrophil mediators in CNS-TB immunopathology. Neutrophils secrete cytokines including IL-1β to enhance neutrophil swarming and recruitment, and TNF-α to promote neutrophil necrosis, granuloma formation and thrombosis leading to ischemia stroke and brain tissue damage. Neutrophils also form NETs containing destructive enzymes which can damage the brain tissue. The release of MMP-9 degrades the extracellular matrix (ECM) resulting in BBB breakdown, leukocytes influx and eventually chronic cerebral inflammation. The ROS production also mediates BBB breakdown and drives neutrophil necrosis. 5-LO: 5-lipoxygenase; AA, arachidonic acid; COX-2, cyclooxygenase-2; CtG, cathepsin G; DC, dendritic cell; LTB4, leukotriene B4; MMP, matrix metalloproteinase; MPO, myeloperoxidase; *M. tb*, *Mycobacterium tuberculosis*; NE, neutrophil elastase; NETs, neutrophil extracellular traps; PGE2, prostaglandin E2; PR3, proteinase 3; ROS, reactive oxygen species; TNF-α, tumor necrosis factor-α. Illustration created with Biorender.com.

The normal brain is devoid of neutrophils, and the recruitment of circulating neutrophils into the CNS during infection is dependent on neutrophil chemoattractants. The contribution of neutrophil mediators to CNS-TB immunopathology is summarized in **Figure 2**. Small bioactive lipid mediators such as prostaglandin E2 (PGE2) and leukotriene B4 (LTB4) generated from arachidonic acid (AA) by cyclooxygenase 2 (COX-2) and 5-lipoxygenase (5-LO) respectively exhibit pro-inflammatory properties and are potent neutrophil chemoattractants (89–93). LTB4 secreted by early-recruited neutrophils function in an autocrine response to induce an exponential neutrophil influx, an effect known as “neutrophil swarming” (94, 95). In addition, LTB4 was demonstrated to induce ICAM-1 expression by vascular endothelial cells to facilitate neutrophil transmigration into the tissue (96). Treatment of *M. tb*-infected C3HeB/FeJ mice with Ibuprofen, an anti-inflammatory drug that inhibits COX-2, strongly suppressed neutrophil recruitment, ameliorated tissue pathology and improved survival, thereby providing further evidence of neutrophils contributing to immunopathology in TB disease (97).

### Neutrophil Cell Death – Protection Versus Pathology

**Figure 3:**
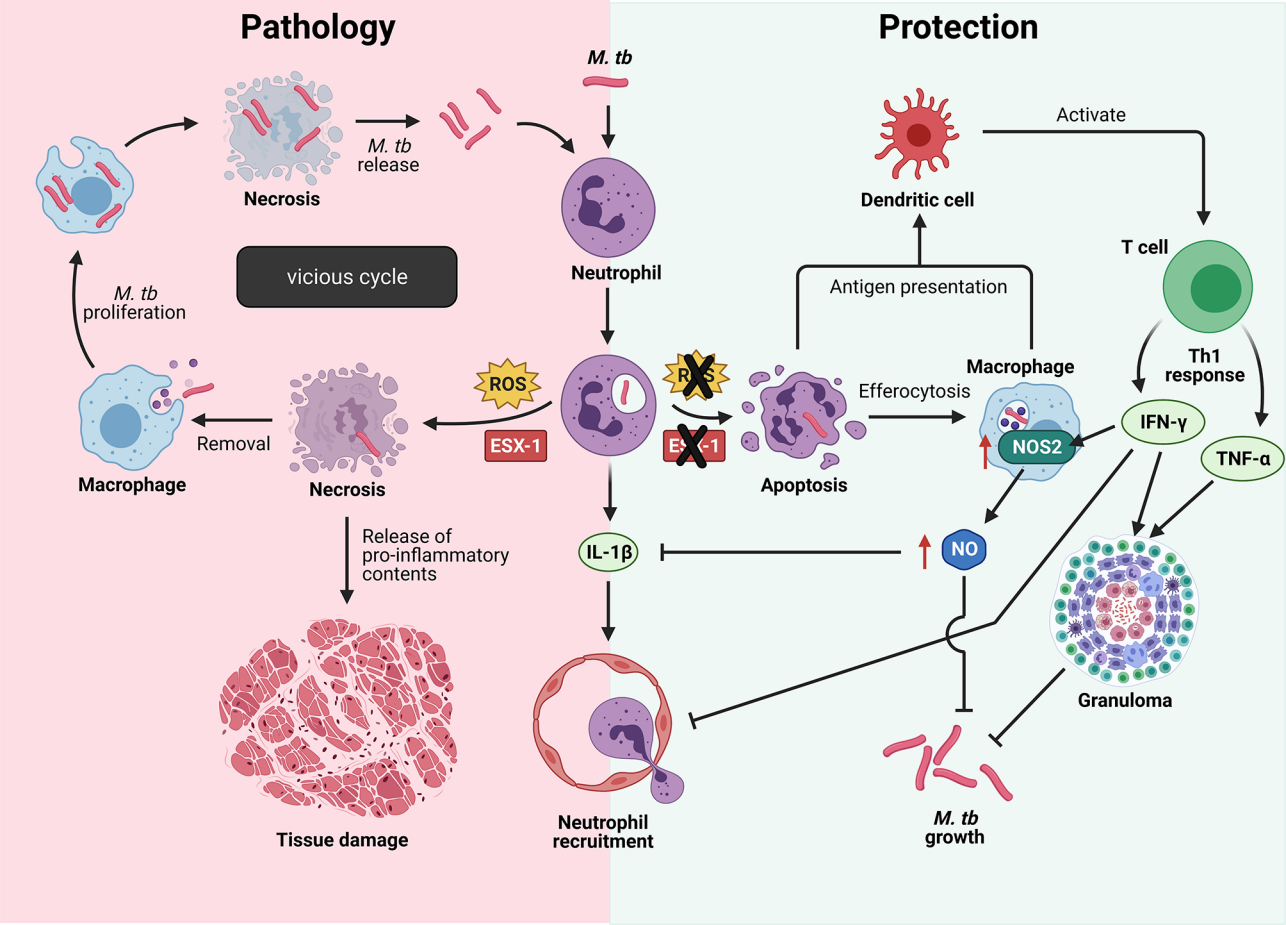
Cell fate of *M. tb*-infected neutrophils determine protection or pathology in TB. Left: Virulent *M. tb* induces neutrophil necrosis in a reactive oxygen species (ROS)- and ESAT-6 secretion system 1 (ESX-1)-dependent manner, resulting in the release of bioactive molecules that damage surrounding host tissue. Removal of necrotic neutrophils by macrophages drive them into necrosis with subsequent release of virulent *M. tb* to infect more host cells, thus resulting in a vicious cycle. Right: When the *M.tb* lacks a functional ESX-1 type 7 secretion system, or the production of ROS by neutrophils is inhibited, neutrophils undergo the default apoptosis instead. Cross-presentation of mycobacterial antigens to dendritic cells result in naïve T cell activation and differentiation into protective Th1 cells that produce interferon-γ (IFN-γ) and tumor necrosis factor-α (TNF-α). IFN-γ-induced nitric oxide (NO) production limits inflammation by inhibiting neutrophil recruitment, thereby reducing immunopathology. IL-1β, interleukin-1β; *M. tb*, *Mycobacterium tuberculosis*; NOS2, nitric oxide synthase 2. Illustration created with Biorender.com.

Neutrophils are the first cells to arrive at the site of *M. tb* infection by migrating along a chemokine gradient formed by IL-8 (also known as CXCL-8) or keratinocyte chemoattractant (KC) in humans or mice respectively (98). Being professional phagocytes, neutrophils rapidly engulf *M. tb*, but the fate of the infected neutrophils and whether they mediate protection or pathology in TB depends on *M. tb* virulence. Human neutrophils fail to kill virulent *M. tb in vitro* due to *M. tb*-induced necrotic cell death, a process that was recently found to be dependent on a functional ESAT-6 secretion system 1 (ESX-1) in *M. tb* and the neutrophil’s own reactive oxygen species (ROS) production (**Figure 3**) (99, 100). Neutrophil necrosis is detrimental to the host as the release of granule proteases and antimicrobial effectors cause damage to neighboring cells and exacerbate tissue damage. In addition, subsequent removal of necrotic neutrophils and virulent *M. tb* by macrophages promote mycobacterial growth, ultimately driving these infected macrophages into necrotic cell death (99). It is likely that the consecutive cycles of infection and host cell necrosis result in TB-associated immunopathology and host tissue damage (101).

In contrast, when neutrophil ROS production is inhibited by pharmacological inhibition of myeloperoxidase (MPO), or when the ESX-1 secretion system is not functional in the attenuated *M. tb* strain, neutrophils undergo apoptosis instead (99). Removal of apoptotic cells, also known as efferocytosis, contributes to host defense in TB. A study has shown efferocytosis of apoptotic neutrophils by macrophages to restore growth control of *M. tb*, as the attenuated *M. tb* ended up in double- or triple-membrane compartment that they could not escape from without a functional ESX-1 secretion system (99). Moreover, efferocytosis of *M. tb*-induced apoptotic neutrophils markedly increased the production of TNF-α by human macrophages (102, 103) and resulted in a decreased viability of intracellular *M. tb* (104). In addition, neutrophils promote the onset of adaptive immunity by delivering *M. tb* to dendritic cells (DCs) in a manner that makes DCs more effective in naïve CD4 T cell activation (86). Upon activation, naïve T cells differentiate into protective Th1 cells that secrete IFN-γ and TNF-α, two cytokines important in the maintenance of granuloma architecture to contain *M. tb* (105–109). Protective immunity during TB requires the host to restrict bacterial growth while limiting inflammation to prevent host tissue damage, and IFN-γ is a key cytokine that serves both functions (110). IFN-γ activates macrophages to kill intracellular mycobacteria and controls inflammation *via* direct and indirect inhibition of neutrophils (110–113). A study has shown IFN-γ to directly inhibit neutrophil accumulation in *M. tb*-infected lung, thereby limiting lung inflammation (110). While IFN-γ-induced nitric oxide (NO) production by murine macrophages have direct anti-mycobacterial activity, NO also limits inflammation by inhibiting IL-1β-dependent neutrophil recruitment (113). These studies highlight the complexity of host-pathogen interactions as well as the crucial role neutrophils play in determining host protection versus pathology in TB disease.

### Neutrophil Extracellular Traps

Neutrophil extracellular traps (NETs) are a meshwork of chromatin fibres coated with cytoplasmic and granule-derived neutrophil antimicrobial peptides and proteases, and NETosis, as the name suggests, refers to the process of NETs release (114). While degranulation and phagocytosis are two long-established antimicrobial functions of neutrophils, NETosis as the third antimicrobial strategy was first described in 2004 (114). NETs allow neutrophils to eliminate pathogens more efficiently by immobilizing the pathogen to prevent its dissemination, and ensuring a high concentration of antimicrobial agents to degrade virulence factors and kill the pathogen (114). Ramos-Kichik et al. demonstrated that *M. tb*-infected neutrophils release NETs which trap mycobacteria but were unable to kill them, suggesting NETs prevent *M. tb* from spreading to other organs while enhancing the local concentrations of released antimicrobial agents against *M. tb* (115).

**Figure 4:**
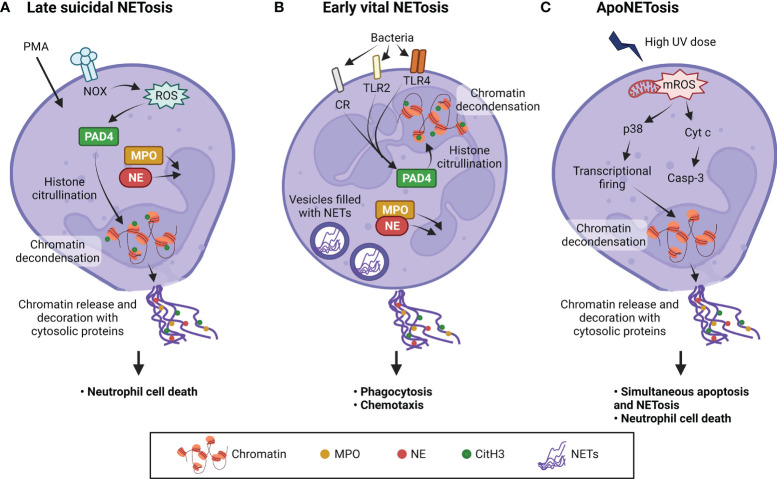
Three types of NETosis induced by different stimuli. **(A)** Stimuli such as phorbol 12-myristate 13-acetate (PMA) induce suicidal NETosis after activating NADPH oxidase (NOX) to produce ROS. **(B)** Vital NETosis is induced within minutes by pathogens such as *Staphylococcus aureus* and *Escherichia coli*, through complement receptors (CR) and Toll-like receptors 2 and 4 (TLR2 and TLR4). This induces protein arginine deiminase 4 (PAD4) activation without the need for oxidants. Citrullination of histones allow chromatin to undergo decondensation and be dispersed in the form of NETs. NE and MPO translocate into the nucleus to promote further unfolding of chromatin. **(C)** ApoNETosis is induced by high-dose ultraviolet (UV) irradiation. This induces large amounts of mitochondrial ROS (mROS), caspase cascade activation, p38 activation, transcriptional firing and NETosis. Under apoNETosis conditions, although both apoptosis and NOX-independent NETosis occur simultaneously, NETotic events predominate apoptotic events. Unlike the other two types of NETosis, PAD4 is not activated and histones are not citrullinated. In addition, nuclear blebbing does not occur unlike classical apoptosis. Casp-3, caspase 3; CitH3, citrullinated H3; Cyt c, cytochrome c; MPO, myeloperoxidase; NE, neutrophil elastase; NETs, neutrophil extracellular traps; ROS, reactive oxygen species. Illustration created with Biorender.com.

Since its discovery in 1996 (116), two types of NETosis have been characterized - late suicidal NETosis and early vital NETosis (**Figure 4**). Late suicidal NETosis, as the name suggests, occurs after several hours of stimulation and is dependent on NADPH oxidase (NOX) production of ROS, whereas early vital NETosis occurs within minutes of stimulation independent of oxidants (117). When NETosis is induced, protein arginine deiminase 4 (PAD4) is activated which converts arginine to citrulline in core histones, resulting in chromatin decondensation necessary for NET formation (118). In suicidal NETosis, NETs are expelled into the extracellular space upon plasma membrane disruption and the neutrophil dies (119), whereas in vital NETosis, NETs are released *via* nuclear envelope blebbing and vesicular export, and the plasma membrane remains intact (120–123). This explains why vital NETosis is mostly associated with bacterial infection, as it prevents the release of phagocytosed bacteria and the neutrophils stay alive to perform other immune functions, such as chemotaxis, phagocytosis, and killing of bacteria (124). NETs induction has been demonstrated in *M. tb*-infected neutrophils (115), but whether the neutrophils undergo vital or suicidal NETosis remains to be investigated. More recently, the finding of a novel form of NOX-independent NETosis that involves both apoptosis and NETosis in the same neutrophil, also known as apoNETosis (125), shows that much investigation remains to elucidate the mechanisms of NETs formation and to better understand the role of neutrophils in physiological and pathological processes.

Transcriptional studies of human TB have been instrumental in unveiling the importance of neutrophils in TB disease (126), but the factors determining disease progression and the molecular mechanisms by which neutrophils drive TB pathogenesis remain poorly understood. A recent mechanistic study by Moreira-Teixeira et al. demonstrated that type I IFN exacerbated disease severity in TB-infected mice by inducing neutrophilic inflammation and NETs formation (127). The presence of NETs in necrotic lung lesions of patients with non-resolving pulmonary TB further supports the clinical relevance of NETs in TB pathogenesis (127). However, the description of NETs in TB is limited to pulmonary TB, and its role remains to be explored in CNS-TB.

Nonetheless, with increasing experimental and clinical evidence of NETs in TB pathogenesis, drugs that manipulate NETs structure or NETosis represent an attractive target for the development of therapeutics. DNase that digests NETs backbone is the oldest and one of the most attractive therapeutic interventions in NETs research (128), but a major limitation with this approach is that NETs-associated histones, proteases and other pro-inflammatory mediators become liberated upon DNA digestion, which can then cause local host tissue damage or systemic inflammation if this happens in the circulation (129, 130). Thus, NETs suppressive drugs that control NETs release without impacting the neutrophils’ antimicrobial activities should be the focus of research moving forward. Screening of 126 compounds by the Palaniyar group to investigate their regulatory effects on NETosis has identified anthracyclines as a class of potent NETosis suppressive drugs (131). Notably, anthracyclines suppress both NOX–dependent and –independent NETs release by inhibiting chromatin decondensation and transcription, while maintaining the neutrophils’ capacity to produce ROS that are crucial for their antimicrobial functions (131). In addition to this, Sollberger et al. also identified a molecule (LDC7559) that inhibits Gasdermin D, a pore-forming protein that punctures granules to release NE, and selectively suppress NETs formation without interfering with neutrophil phagocytosis (132). These selective pharmacological inhibitors of NETs formation present a promising avenue of research to be further explored.

*M. tb*-activated neutrophils also release heat shock protein 72 (Hsp72), a stress-induced protein that binds to NETs to trigger the secretion of pro-inflammatory cytokines TNF-α and IL-1β from adjacent macrophages (14). Apart from their pro-inflammatory and antimicrobial functions, NETs also provide a stimulus and scaffold for thrombus formation which leads to ischemic strokes and permanent brain damage, although the role of NETs in thrombosis have not been demonstrated in the context of TB (7, 15, 16).

### Neutrophil Pathogenic Enzymes and ROS

During *M. tb* infection, neutrophils release NETs containing histones, MPO, MMPs and serine proteases such as neutrophil elastase (NE), proteinase 3 (PR3), and Cathepsin G (CtG) that may result in tissue damage (114, 115, 118, 133). Using a gene-targeted approach, Guyot et al. demonstrated that neutrophil serine proteases (NSPs) CtG, PR3 and NE synergistically caused more tissue destruction than NE alone (133). Additionally, these NSPs increased the activity of other tissue-destructive proteases such as macrophage elastase (MMP-12) and gelatinase B (MMP-9) (133). MMPs, in particular MMP-9, play important roles in brain ECM degradation, resulting in BBB breakdown and brain tissue damage. In the analysis of human brain biopsy specimens, high concentration of neutrophils expressing MPO were present in the necrotic zone of the granuloma and within areas of brain infarction of TBM patients (47). The staining of NE and MMP-9 were also observed in CNS-TB human brain biopsies (9). Moreover, the whole-blood transcriptomic data of TBM-immune reconstitution inflammatory syndrome (IRIS) patients revealed an increase in neutrophil-dependent inflammatory response with significantly more neutrophil-associated transcripts including MPO, MMP-8 and -9, CtG, lipocalin 2 (LCN2) and α-defensin (DEFA1/3/4) compared to TBM non-IRIS (134). All these studies implicate neutrophils in driving immunopathology in CNS-TB.

*M. tb*-induced ROS production by neutrophil MPO has been shown to drive human neutrophils into necrotic cell death and directly damage the vascular endothelium and brain parenchyma (100, 135). ROS causes loss of endothelial barrier integrity by downregulating the expression of tight junction proteins, as well as inducing a shift in the membrane localization of tight junction proteins to the cytoplasm, thus increasing BBB permeability (136). A compromised BBB facilitates leukocyte transmigration into the CNS as well as influx of plasma resulting in edema. In addition, ROS disrupts the cadherin-β-catenin complex, which results in adherens junction disassembly at cell-cell contact, further contributing to BBB disruption (137).

### Neutrophil Cytokines and Chemokines in CNS-TB Immunopathology

Activated neutrophils secrete a range of cytokines and chemokines to signal other innate and adaptive immune cells. For example, CCL-2, -3 and -20 recruit monocytes, CCL-17 recruits dendritic cells (DCs), and IL-6, TNF-α and IL-23 attract T lymphocytes to the site of infection (138). The concentration of CXCL-8, a predominant neutrophil stimulatory and chemotactic chemokine, was found to be increased in the CSF of TBM patients (139). Immunohistochemical staining showed that IL-2 and IL-17A, cytokines that enhance the recruitment of neutrophils and which have a role in pulmonary TB (140), were distributed intracellularly in the granulomas of TBM patients, contributing to a pathological inflammatory response (47). The finding of significantly increased CSF IL-17A concentrations in TBM-IRIS patients associated with severe CNS inflammation further supports a detrimental role for IL-17A in CNS-TB (141).

Neutrophils produce TNF-α, the key cytokine involved in the initiation of immune response to *M. tb* and in the long-term control of infection. Not only is TNF-α important for macrophage activation and recruitment to the site of infection, TNF-α is also critical for granuloma formation and architecture to contain *M. tb* (142). In keeping with the protective role of TNF-α against *M. tb* infection, intracerebral *M. tb*-infected TNF^-/-^ mice demonstrated an increased infiltration of leukocytes into the brain (79, 143, 144), which may be explained by the inability of TNF^-/-^ mice to effectively control *M. tb*, thereby resulting in an uncontrolled recruitment of immune cells into the CNS. While TNF-α confers protective immunity against *M. tb*, it also causes immunopathology by increasing BBB permeability (73). In addition, TNF-α and IL-17 have demonstrated synergistic pro-coagulant and pro-thrombotic effects on vessels (145), potentially contributing to ischemia stroke. Given the potential protective and pathological roles of TNF-α in CNS-TB, a “Goldilocks phenomenon” may exist where a certain amount of TNFα is required for the human host but excess is deleterious.

## Matrix Metalloproteinases and Their Role in CNS-TB

MMPs are zinc-containing enzymes that degrade extracellular matrix at a neutral pH. The MMP family comprises 25 related but distinct proteases of which 24 are found in mammals. In addition to ECM degradation, they have key functions in wound healing, angiogenesis, inflammation and host defense (146). MMPs can be broadly classified into several sub-families on the basis of substrate specificity, namely gelatinases, collagenases, stromelysins, matrilysin, metalloelastase and the membrane-type metalloproteinases (**Table 2**). While the classical ECM substrates include collagen, elastin and fibronectin, recent work has identified a variety of non-matrix substrates including adhesion proteins (157, 158), receptors (159), cytokines and chemokines (160–163). There is considerable overlap in MMP substrates, especially among the ECM proteins (164). Although the shared substrate potential may appear as a form of biochemical redundancy, the selectivity of MMP catalysis is regulated by enzyme affinity and compartmentalization (146). Kinetic studies have demonstrated that specific MMPs degrade some substrates more efficiently than others do. For example, MMP-2 and -9 degrade gelatin more efficiently than other MMPs (165). Compartmentalization, which is the pericellular accumulation of MMPs, allow MMPs to target specific substrates in the pericellular space for catalysis (146). Cells do not indiscriminately release MMPs as that would result in non-specific proteolysis and tissue injury. Several reports have demonstrated specific interactions between MMPs and cell membrane anchors such as MMP-2 binding to α_v_β_3_-integrin (166), MMP-9 to CD44 (159) and MMP-7 to surface proteoglycans (167, 168). This enables MMPs to accumulate to high concentrations locally and thus increase substrate specificity and catalysis efficiency.

**Table 2:** Matrix metalloproteinases, substrates and their activating capacity.

Enzyme	Common name	Proposed substrate	Activator of
**Gelatinases**			
MMP-2	Gelatinase A (72-kDa gelatinase)	Gelatin, collagen I, **IV**, V, VII, X, XI, XIV, gelatin, aggrecan, versican, proteoglycan link protein, tenascin, **fibronectin**, **laminin**, laminin-5, fibrillin, elastin, vitronectin, α_2_-M, latent TNF, MBP, α_1_-AT, neurocan, IL-1β precursor, CXCL-12, CCL-7, occludin, claudin-5	MMP-9, -13
MMP-9	Gelatinase B (92-kDa gelatinase)	Gelatin, **collagen IV**, V, VII, X, XIV, aggrecan, versican, proteoglycan link protein, **fibronectin**, **nidogen**, elastin, vitronectin, α_2_-M, latent TNF, latent TGF-β, latent VEGF, fibrin, NG2 proteoglycan, MBP, α_1_-AT, IL-1β precursor, occludin, claudin-5, ZO-1	ND
**Collagenases**			
MMP-1	Collagenase-1 (fibroblast collagenase)	Collagen I, II, III (III>I), VII, VIII, X, gelatin, aggrecan, versican, proteoglycan link protein, L-selectin, **nidogen**, tenascin, serpins, α_2_-M, latent TNF, MBP, α_1_-AT	MMP-2, -9
MMP-8	Collagenase-2 (neutrophil collagenase)	Collagen I, II, III (I>III), V, VII, VIII, X, gelatin, aggrecan, **fibronectin**, serpins, α_2_-M, α_1_-AT	ND
MMP-13	Collagenase-3	Collagen I, II, III (II>I or III), **IV**, IX, X, XIV, gelatin, aggrecan, **perlecan**, **fibronectin**, **laminin**, tenascin, fibrillin, serpins	MMP-2, -9
**Stromelysins**			
MMP-3	Stromelysin-1	Collagen III, **IV**, V, IX, X, gelatin, versican, aggrecan, proteoglycan link protein, **perlecan**, **fibronectin**, **laminin**, tenascin, elastin, fibrillin, latent TNF, latent TGF-β, MBP, α_1_-AT, antithrombin-III, IL-1β precursor, occludin, claudin-5, VE cadherin	MMP-1, -8, -9, -13
MMP-10	Stromelysin-2	Collagen III, **IV**, V, gelatin, **nidogen**, aggrecan, proteoglycan link protein, **fibronectin**, elastin	MMP-1, -7, -8, -9
MMP-11	Stromelysin-3	α_1_-AT, **fibronectin**, **laminin**	ND
**Membrane-type (MT) MMPs**			
MMP-14	MT1-MMP	Collagen I, II, III, gelatin, aggrecan, **fibronectin**, **laminin**, tenascin, vitronectin, fibrillin	MMP-2, -13
MMP-15	MT2-MMP	Aggrecan, **fibronectin**, **laminin**, fibrin	MMP-2, -13
MMP-16	MT3-MMP	Gelatin, casein, fibrin, syndecan-1	MMP-2
MMP-17	MT4-MMP	Gelatin, latent TNF	MMP-2
**Other MMPs**			
MMP-7	Matrilysin	**Collagen IV**, X, gelatin, aggrecan, proteoglycan link protein, **fibronectin**, **laminin**, **nidogen**, vitronectin, pro-α defensins, FAS ligand, latent TNF, syndecan-1, E-cadherin, elastin, MBP, α_1_-AT	MMP-2, -9
MMP-12	Metalloelastase (macrophage elastase)	**Collagen IV**, gelatin, aggrecan, **fibronectin**, **laminin**, fibrillin, elastin, vitronectin, latent TNF, MBP, α_1_-AT	ND

Substrates in the brain vascular basement membrane are in bold, while substrates in the brain parenchymal ECM are underlined. α_1_-AT, α_1_-antitrypsin/α_1_-proteinase inhibitor; α_2_-M, α_2_-macroglobulin; MBP, myelin basic protein; ND, not determined; TGF, transforming growth factor; TNF, tumor necrosis factor; VEGF, vascular endothelial growth factor; ZO-1, zona occludens 1. Adapted from (146–156).

The catalytic activity of MMPs are regulated by pro-enzyme activation and endogenous inhibitors such as α_2_-macroglobulin in the plasma and tissue inhibitors of metalloproteinases (TIMPs) in the tissue (169). While there are 24 mammalian MMPs, only four TIMPs (TIMP-1 – 4) have been identified that inhibit MMP activity by binding to their catalytic site in a 1:1 molar stoichiometry (170, 171). Similar to MMPs having variable substrate affinities, TIMPs also differ in their affinities for specific MMPs. Deciphering the activity of MMPs is made more challenging by the fact that MMP-TIMP interaction does not always lead to inhibition, as exemplified by the need for TIMP-2 to bind MMP-2 in a complex with membrane-bound MMP-14 to induce MMP-2 activation (146).

**Figure 5:**
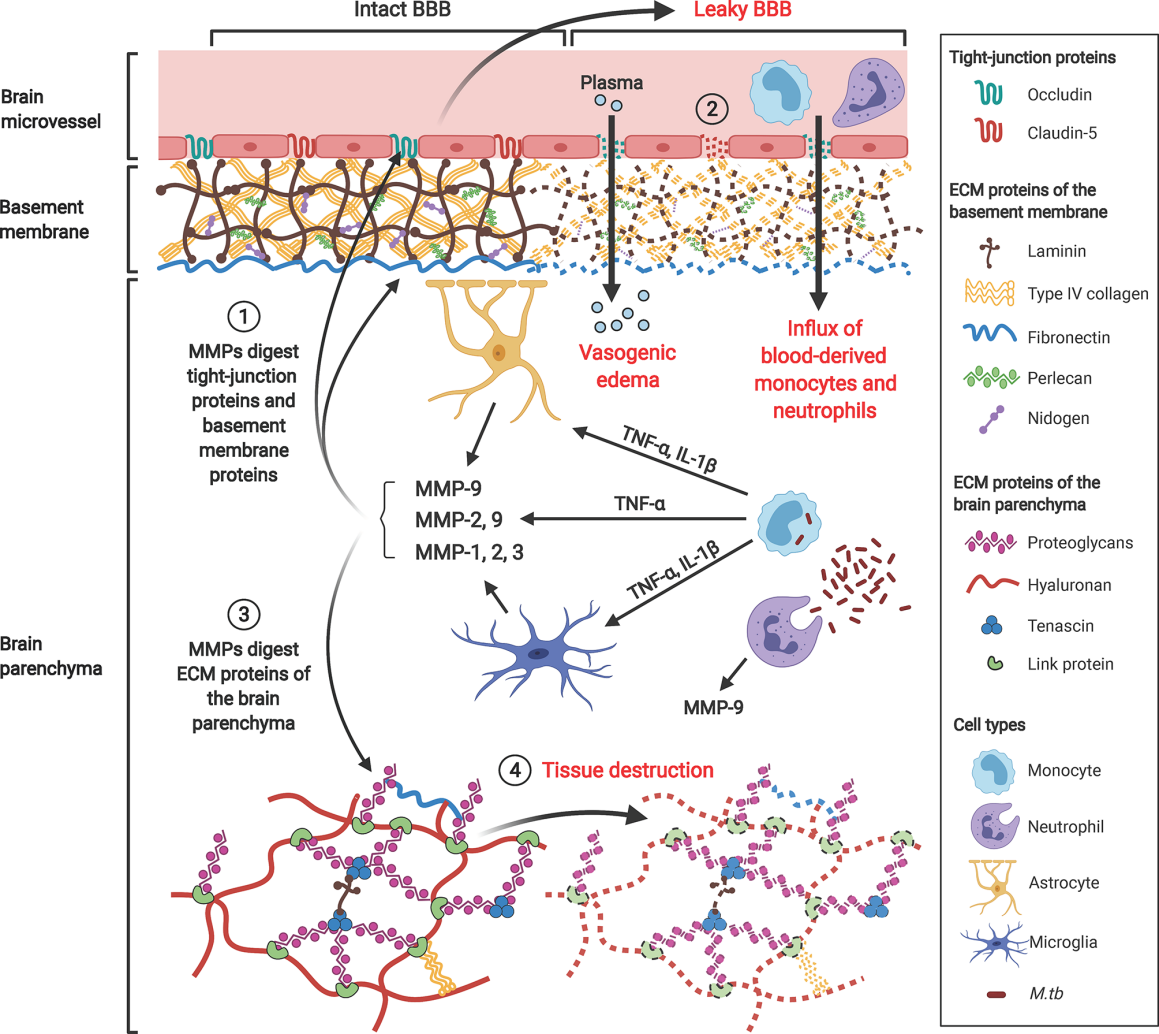
Gelatinases MMP-2 and -9, collagenase MMP-1 and stromelysin MMP-3 contribute to BBB breakdown and brain tissue damage in CNS-TB. *M. tb*-infected monocytes interact with astrocytes and microglia in the brain to induce their secretion of MMP-9, -1 and -3 respectively, a process that is driven by pro-inflammatory mediators TNF-α and IL-1β. ① These MMPs degrade TJPs occludin and claudin-5 and ECM proteins of the basement membrane including type IV collagen, laminin, nidogen, perlecan and fibronectin. ② This BBB breakdown drives influx of plasma resulting in vasogenic edema and facilitates further influx of circulating inflammatory cells such as monocytes and neutrophils into the brain. ③ MMPs also degrade proteoglycans (aggrecan, versican, brevican), proteoglycan link proteins and tenascins found within the ECM of the brain parenchyma, thus resulting in ④ brain tissue destruction adding to the cerebral inflammatory response. MMP-9 secreted from neutrophils further compromise the BBB and exacerbate tissue damage. Illustration created with Biorender.com.

Excessive MMPs degrade the brain ECM, leading to BBB breakdown, plasma leakage and brain tissue destruction in CNS-TB (**Figure 5**). The major MMPs that disrupt the BBB basement membrane are gelatinases MMP-2 and -9, due to their ability to degrade type IV collagen, the main extracellular matrix of the BBB. Under normal brain physiological conditions, MMP-2 is constitutively expressed in large amounts and can be found in the CSF (172). Conversely, MMP-9 is normally absent or present at low levels in the normal brain, and is upregulated during inflammation (173) or infection (174, 175). Neutrophils may be the main cellular source of MMP-8/9 (7, 9). Our work demonstrated that MMP-9-expressing neutrophils were present in tuberculous granulomas in CNS-TB and neutrophil-derived MMP-9 secretion was upregulated by *M. tb* (9). Apart from type IV collagen, the gelatinases are also capable of degrading laminin, fibronectin, nidogen, versican, aggrecan, tenascin and proteoglycan link protein found within the cerebral vascular basement membrane and parenchymal ECM (147). Additionally, MMP-2 and -9 have been demonstrated to degrade TJPs occludin and claudin-5, thereby further increasing BBB permeability (148). Thus, gelatinases MMP-2 and -9 likely play key roles in mediating BBB breakdown and tissue destruction in the brain. A compromised BBB results in plasma leakage into the CNS, causing vasogenic edema and further facilitates the influx of circulatory inflammatory cells into the brain.

In organs like the brain, interactions between different cellular networks influence the eventual MMP expression, which in turn define the final pathophysiological consequence. We have previously demonstrated that astrocytes and microglia secrete MMP-9, -1 and -3 respectively in response to *M. tb*-infected monocytes but not upon direct *M. tb* infection (176, 177). This network-dependent secretion of MMPs is driven by pro-inflammatory mediators TNF-α and IL-1β. In addition to its role as an activator of MMP-9, a recent study has shown the importance of MMP-3 in BBB breakdown by reducing tight junction and VE-cadherin proteins in brain microvascular endothelial cells (149). LPS-activated microglia have significantly reduced TNF-α in the presence of a broad spectrum MMP inhibitor BB94 (178), while a separate study similarly reported that inhibition of MMP-3 or -9 resulted in a suppression of iNOS, IL-1β, IL-1Ra and IL-6 gene expression, and TNF-α at the post-transcriptional level in activated microglia (179). Thus, MMPs may influence cytokine secretion in microglia, suggesting a potential effect of neutrophil-derived MMPs in mediating microglia’s cytokine responses during CNS-TB.

Although many studies have focused on the contribution of MMPs to host immunopathology, there is increasing evidence to show that these proteases also play a crucial role in granuloma formation (180). Several MMPs have been shown to cleave and modulate the functions of cytokines and chemokines such as IFN-γ, IL-1β, TNF-α, CXCL-8 and CCL-7, thereby regulating chemokine gradients and leukocyte recruitment to the site of infection (146). Treatment of pulmonary TB mouse models with batimastat, a broad-spectrum MMP inhibitor, resulted in a delayed induction of granuloma or the formation of smaller granulomas with increased collagen content (181, 182), but the same has not been shown for CNS-TB. Consistent with these findings, MMP-9-deficient mice demonstrated a reduced recruitment of macrophages leading to the development of smaller granulomas (183). Thus, while MMPs are believed to degrade ECM substrates as their primary function, some MMPs, such as MMP-9, may also have dual roles in tissue remodeling and ECM deposition (180).

### MMP Studies in Human CNS-TB and the Gaps in Knowledge

Research into BBB disruption in human CNS-TB is limited, but several studies have lent evidence on the pathogenic role of MMPs in CNS-TB (10, 184–187), and that MMPs may drive BBB breakdown and CNS-TB immunopathology. Specific MMPs and TIMPs in the CSF and systemic circulation of TBM patients may provide an indication of the overall MMP activity *in vivo* (184, 186) and may be a surrogate of BBB breakdown. However, the current gap is the lack of direct association of MMP concentrations and an accepted index of BBB breakdown (10, 188, 189).

Numerous studies have found CSF MMP-9 concentrations in TBM patients to be upregulated, with MMP-9 upregulation associated with disease severity (190), neurological complications (184, 186), and brain tissue damage (185, 186). Another member of the gelatinase sub-family MMP-2 was also implicated in TBM pathogenesis. Patients with subacute meningitis, including fungal meningitis and TBM, had higher CSF MMP-2 and -9 as well as TIMP-1 (but not TIMP-2) than patients with non-inflammatory neurological diseases. Increased MMP-9 correlated with CNS complications including depressed consciousness and psychiatric symptoms (185). Furthermore, the upregulated MMP-2 and -9 concentrations in CSF persisted late into the course of TBM (187), indicating that anti-tuberculous treatment (ATT) is ineffective in reducing gelatinase MMP-2 and -9 concentrations. The inability of standard ATT to mitigate the increased MMP-2 and -9 concentrations in TBM patients indicates that there is an urgent need to explore adjunctive treatment to suppress these pathogenic MMPs. A study conducted by Green et al. found that adjunctive dexamethasone, the standard of care in CNS-TB, significantly decreased CSF MMP-9 concentrations in TBM patients, but the decline in MMP-9 concentrations was not associated with improved outcome (10). The concentration of MMPs should be considered in relation to their specific endogenous TIMPs, as the balance between MMP and TIMP concentrations determine the overall MMP activity, thereby influencing TBM pathogenesis and patient outcome. 20 years ago, our group showed for the first time a significant increase in MMP-9 but not TIMP-1 in the CSF of TBM patients, suggesting a matrix-degrading phenotype in TBM where MMP-9 activity was relatively unopposed by TIMP-1 (186). In addition, the imbalance of MMP-9:TIMP-1 ratio correlated with mortality and neurological morbidity (such as unconsciousness, confusion and neurological deficits) in TBM patients (186).

However, the association between increased MMP-2 and -9 concentrations and poor patient outcome is not consistent, and several studies have demonstrated no association between MMP concentrations and outcome (188, 189, 191, 192). This is unsurprising as MMPs, specifically MMP-2, -3, and -9, also play important roles in normal brain development. There is increasing evidence that MMPs perform diverse functions, both protective and pathological, at different concentrations, in different age groups and at different time points of infection (193), which may explain these differing observations. In a recent study of 40 TBM patients by Mailankody et al., CSF-serum albumin index was used as an indicator of BBB permeability, but there was no association between CSF MMP-9 and the CSF-serum albumin index. Both MMP-9 and TIMP-1 were also not associated with treatment outcome. In contrast, a significant positive correlation between MMP-9 levels and Glasgow coma scale (GCS) was found, indicating that higher MMP-9 concentrations were associated with a favourable outcome (188). In a study of pediatric TBM infections, higher MMP-9 concentrations were found to be associated with a good outcome, possibly due to the role of MMP-9 in recovery and ongoing neurodevelopment, including angio- and myelino-genesis, synaptic plasticity and the growth of axons (189). Thus, there is conflicting literature on whether MMP-9 is protective or pathogenic which is likely to reflect diverse patient populations.

**Figure 6:**
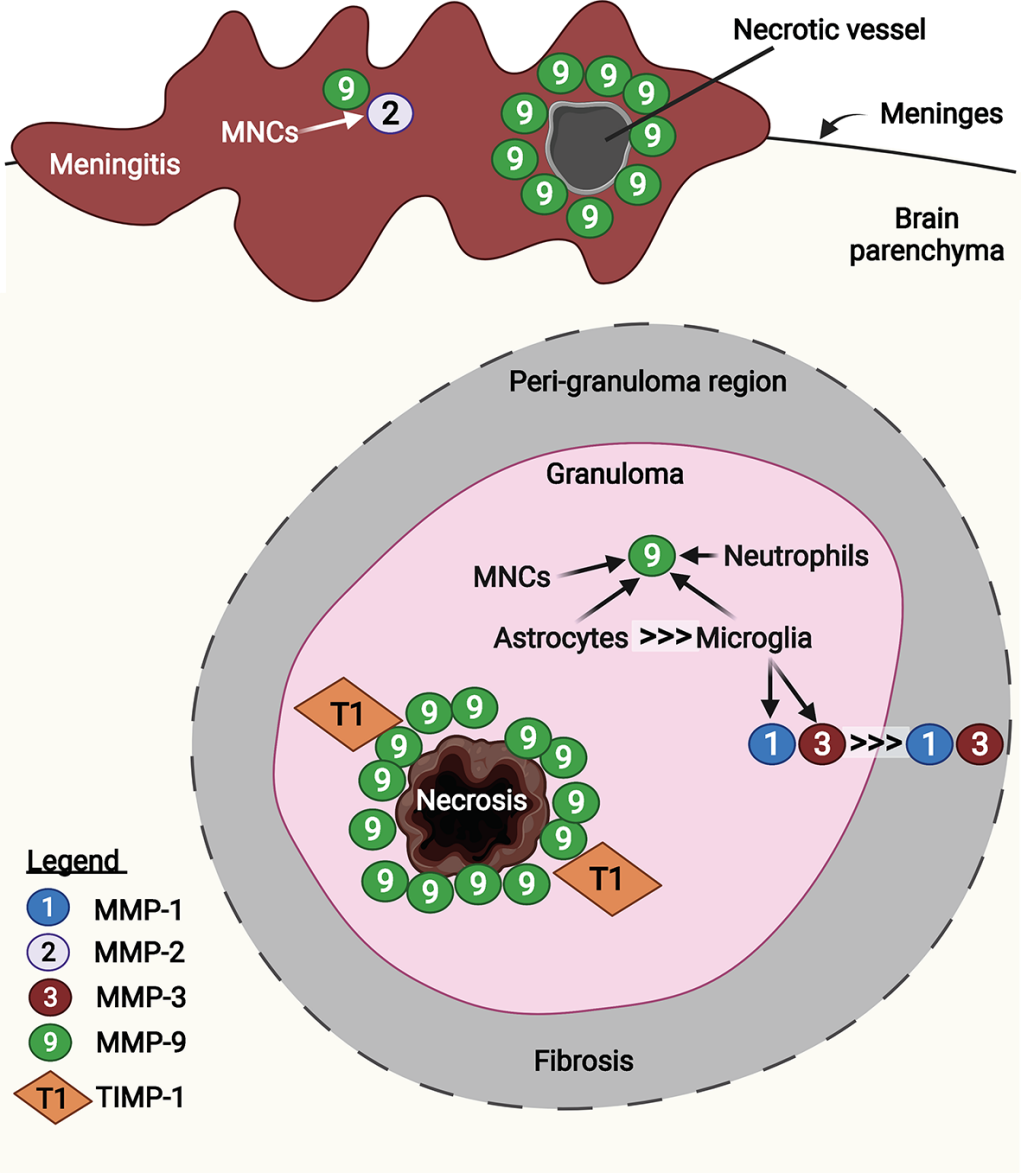
MMP-1, -2, -3, and -9 are expressed in human CNS-TB granulomas. Infiltrated mononuclear cells (MNCs) in the meninges were immunoreactive for MMP-2 and -9. In particular, MMP-9 was expressed in the perivascular leukocytes at necrotic vessel, contributing to BBB disruption (185). In the granuloma, MMP-9 was highly expressed around the area of caseous necrosis, unopposed by TIMP-1 (194). Astrocytes are the main CNS cellular source of MMP-9, compared with other sources including MNCs, neutrophils and microglia (184). Microglia-derived MMP-1 and -3 were found decreasing towards fibrosis peri-granuloma region (176). Illustration created with Biorender.com.

TB granulomas (tuberculomas), which occur frequently in CNS-TB, demonstrate high expression of several MMPs including MMP-1, -2, -3 and -9 (**Figure 6**) (9, 176, 184, 185). Immunohistochemical analysis of brain biopsies from CNS-TB patients demonstrated that MMP-2 and -9 exhibit distinct localization within the brain granuloma (185). While infiltrating mononuclear cells in the meninges demonstrated immunoreactivity for both MMP-2 and -9, mononuclear cells that infiltrated into the brain parenchyma were immunoreactive for MMP-9 but not MMP-2 (185). In meningeal vessels that showed necrotic changes, MMP-9 was expressed in the perivascular leukocytes, thereby providing evidence that MMP-9 is associated with BBB disruption in TBM *in vivo* (185). The role of MMP-9 in brain tissue damage was corroborated by the high MMP-9 expression around the area of caseous necrosis in TB granuloma, which was relatively unopposed by the presence of few TIMP-1-positive stromal cells (194). Harris et al. also demonstrated upregulated MMP-9 secretion in astrocytes near CNS-TB granulomas and downregulated TIMP-1 expression in the brain tissue of CNS-TB patients (184). Additionally, although both astrocytes and tissue-resident macrophages and microglia expressed high MMP-9 in the CNS-TB brain tissue, macrophages and microglia were present as much lower numbers than the astrocytes, indicating astrocytes as the major CNS cellular source of MMP-9 in CNS-TB (184). By immunostaining for MMP-9 and neutrophil elastase, we demonstrated the presence of MMP-9 secreting neutrophils in CNS-TB granulomas (9). In addition to MMP-9, MMP-1 and -3 secretion were also found to be highly expressed in the center of granuloma which decrease towards the fibrotic, peri-granuloma region (176). This increased expression of MMP-1 and -3 were associated with microglia in the granuloma and peri-granuloma region and p38-positive microglia infiltrating necrotizing CNS-TB granulomas (176, 195). Collectively, these results support a role for MMPs, in particular MMP-1, -3, and -9, in brain tissue destruction in CNS-TB patients. However, the contribution of MMP-2 to BBB breakdown and tissue damage in CNS-TB remains further evaluation, as TNFα has been shown to suppress microglial MMP-2 secretion by *M. tb*-infected monocyte-dependent networks (195).

Stromelysin MMP-10 is functionally important in TB. It was found upregulated in induced sputum and bronchoalveolar lavage fluid from TB patients compared to respiratory symptomatic controls (196). The inhibition of MMP-10 activity has shown to decrease DQ collagen degradation by *M. tb*-infected macrophages, and its upregulation in macrophages was induced by virulent *M. tb* in Early Secretory Antigenic Target-6 (ESAT-6)-dependent manner (196). Rohlwink et al. proposed that MMP-10 and IL-17-augmented MMP-3 (140) may activate MMP-1 to propagate collagenase activity in cellular networks (193). *M. tb* stimulation of peripheral blood mononuclear cells (PBMCs) from TB-IRIS patients also found with elevated MMP-1, -3, -7, and -10 protein secretion compared to non-IRIS controls (197). Similarly, the concentrations of CSF MMP-1, -7, and -10, together with neutrophil-associated mediators were found higher in TBM-IRIS than non-IRIS controls (141). However, the functional role of MMP-10 in CNS-TB, and whether it is associated with MMP-3 and neutrophils and their mediators remains an open question.

## Neutrophils and Strokes in CNS-TB

Ischemic stroke is a devastating complication found in 15- 67% of TBM patients, associated with poor outcome and higher mortality compared to those without stroke (198, 199). A higher incidence of stroke was reported in younger children and those with advanced stages of TBM (200). CSF white cell count and basal meningeal enhancement were identified as independent risk factors for stroke in young TBM patients (201). Most of the strokes in TBM are multiple, bilateral and located in the basal ganglia (198, 199). The extensive damage of cerebral vessels or vasculitis contributed to widespread infarctions in TBM patients (202), which was significantly associated with hydrocephalus (203). Dysregulated inflammation is likely to contribute to TBM-related stroke. The upregulated inflammatory cytokines TNF-α, MIP-1α, IL-6, IL-8, IL-4 and IL-1β concentrations in CSF samples were correlated with the presence of infarcts in TBM patients (204, 205). In another study, the concentrations of lipocalin-2, soluble receptor for advanced glycation end products (sRAGE) and CXCL10 were significantly higher in the CSF of children with TBM-related stroke compared to TBM without stroke (206). Moreover, Schoeman et al. reported a prothrombotic profile of TBM children, with increased procoagulant factor (Factor VIII) expression and decreased in both anticoagulant Protein S expression and fibrinolytic activity (207). These findings also indicated a hypercoagulable state in TBM which is more pronounced in stage III of TBM with increased risk of thrombosis and infarction (207). Platelets are likely to be key in the inflammatory and thrombotic response to CNS-TB (60).

Since its first description in 2004, NETs have been implicated in many non-infectious diseases associated with thrombosis, including diabetes mellitus, autoimmune diseases, atherosclerosis, vasculitis, and thrombosis (117). *In vivo*, NETs are degraded by plasma deoxyribonucleases (DNases) to facilitate their subsequent clearance by macrophages (208–210). Mice deficient in DNase-1 and -3 die within several days after neutrophil activation due to blood vessel occlusion by intravascular NETs, suggesting a role for NETs in clot formation (208). The presence of citrullinated histone 3 (CitH3), a NETosis marker, in the thrombi of mice and humans; and the finding that mice form smaller thrombi when treated with DNase, further support this hypothesis (211–214). The clinical importance of NETs in thrombosis is highlighted in the study by Ducroux et al., where they demonstrated, through histological analysis, that NETs structures were concentrated in the outer layers of patient-derived ischemic stroke thrombi, and that *ex vivo* thrombolysis was accelerated when DNase-1 was added to the standard tissue plasminogen activator (16). Another study that analysed 86 thrombi from ischemic stroke patients undergoing endovascular treatment supported this finding (215). The mechanisms by which NETs stimulate thrombus formation and NETs-induced coagulation remains to be fully dissected.

**Figure 7:**
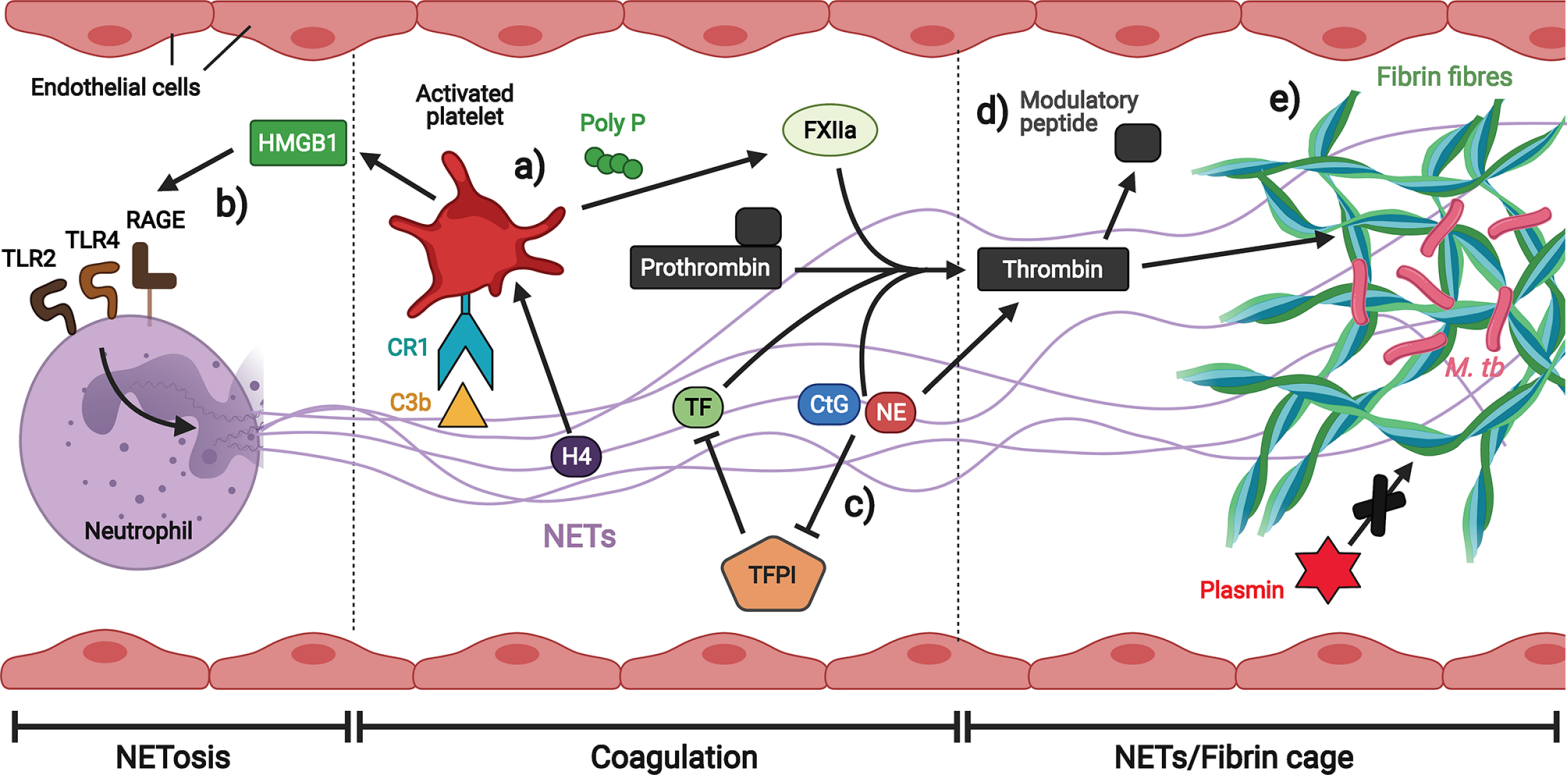
Neutrophil extracellular traps (NETs) promote thrombosis in brain microvessels. **(A)** Platelets interact with C3b and histones on NETs to stimulate the secretion of polyP which activates the extrinsic coagulation pathway. **(B)** Platelet-derived HMGB1 induces NETosis. **(C)** Multiple components of NETs induce coagulation either directly or by inhibiting the extrinsic coagulation pathway inhibitor. **(D)** NE is able to generate thrombin-derived immune modulatory peptides. **(E)** Fibrin fibres strengthened by NETs immobilize *M. tb* and are less prone to degradation by plasmin. Illustration created with Biorender.com.

There may be synergistic interaction between NETs generated in response to TB infection and platelets in thrombosis (**Figure 7**) (216). While NETs promote thrombin generation (217), activated platelets, in turn, trigger NETs formation (218). During thrombosis, hypoxia-induced release of von Willebrand factor (vWF) and p-selectin from the endothelium recruits and activates neutrophils, initiating NETs production (219, 220). Platelets interact with C3b and histones on NETs to stimulate the secretion of polyP, a compound that activates the extrinsic coagulation pathway by activating factor XII (FXIIa) (221–224). In addition, histone 4 (H4) on NETs activates platelets to secrete high mobility group box 1 protein (HMGB1), a damage associated molecular pattern (DAMP) that stimulates NETosis by RAGE (Receptor for Advanced Glycation End products), TLR2 and TLR4 receptors, thereby creating a positive feedback loop (212). In general, NETs associated with platelets serve as a scaffold on which thrombi can form, and several components of NETs have been shown to induce fibrin generation. Tissue factor (TF) induces thrombin cleavage directly (225), while the negatively charged nucleic acids bind and activate FXII (213). NETs-associated serine proteases NE and CtG also contribute to fibrin formation on NETs by degrading tissue factor pathway inhibitor (TFPI), the main extrinsic coagulation pathway inhibitor (226). CtG has also been demonstrated to proteolytically activate platelet receptors to enhance platelet accumulation (227). In addition, NE cleaves prothrombin to generate small antibacterial peptides that exert immunomodulatory effects, thereby providing a logical rationale for the induction of coagulation by NETs: the generation of antibacterial molecules as a by-product of coagulation for host defense (15). The fibrin clots can also serve an antimicrobial function by strengthening NETs structure to immobilize pathogens and limit their spread.

Correlative light and electron microscopy (CLEM) images of NETs and fibrin fibrils in lung tissue sections of legionnaire’s pneumonia provide evidence that both structures are interwoven (228–230). However, interwoven NETs/fibrin structures form a scaffold that entraps platelets and red blood cells (RBCs) (213, 231) and are more resistant to plasmin-mediated fibrinolysis, which may contribute to pathology in several thrombosis-related diseases (232), including strokes in CNS-TB, although this has not been proven. Further investigation on the interaction between NETs and platelets in the context of CNS-TB to uncover their potential immunopathology mechanisms are warranted.

## Adjunctive Therapy for CNS-TB

The recommended anti-tuberculous treatment regime for CNS-TB was largely extrapolated from the principles of treatment for pulmonary TB, as no clinical trial has managed to establish the optimal therapy for CNS-TB (39). While the drug composition and dosing of treatment regime is the same as for pulmonary TB, treatment duration for CNS-TB is extended to 10 to 12 months (233). The first 2 months of intensive phase of treatment uses a 4-drug combination of rifampin (RIF), isoniazid (INH), pyrazinamide (PZA), and ethambutol (EMB), followed by a 2-drug combination of RIF and INH in the continuation phase (234). With respect to the efficacy of ATT drugs in crossing the blood-brain barrier (BBB), INH and PZA showed excellent CSF penetration, while RIF and ETH have limited CSF penetration (233, 235). Although CSF concentration of RIF is only 10% of plasma, which barely exceeds the minimum inhibitory concentration (MIC) against *M. tb*, the high mortality associated with RIF-resistant TBM affirms its key role in CNS-TB treatment (236).

Several clinical trials have evaluated the efficacy of a high-dose RIF as an intensified ATT regime for TBM, but have met with contradicting results (237–239). The use of high-dose intravenous (i.v.) RIF (13mg/kg) for the first 2 weeks significantly reduced mortality at 6 month by about 50% (237). In contrast, there was no association between high-dose RIF treatment (15mg/kg) and improved survival at 9 months (238). Another study which increased the RIF dosage to 30 mg/kg found no significant difference in 6-month mortality between the 10-, 20- and 30 mg/kg oral RIF treatment arms (239).

Nonetheless, clinical outcome of CNS-TB patients are often poor even with ATT. Long-term neurological deficits occur in 5 to 40% of surviving TBM patients, and studies that performed repeated MRI/CT scans in TBM patients on ATT reported frequent worsening of radiological findings (66, 240–243). This signifies a need to develop new interventions to improve outcome, which led to an increased research interest in repurposing existing drugs as adjunctive therapies.

**Figure 8:**
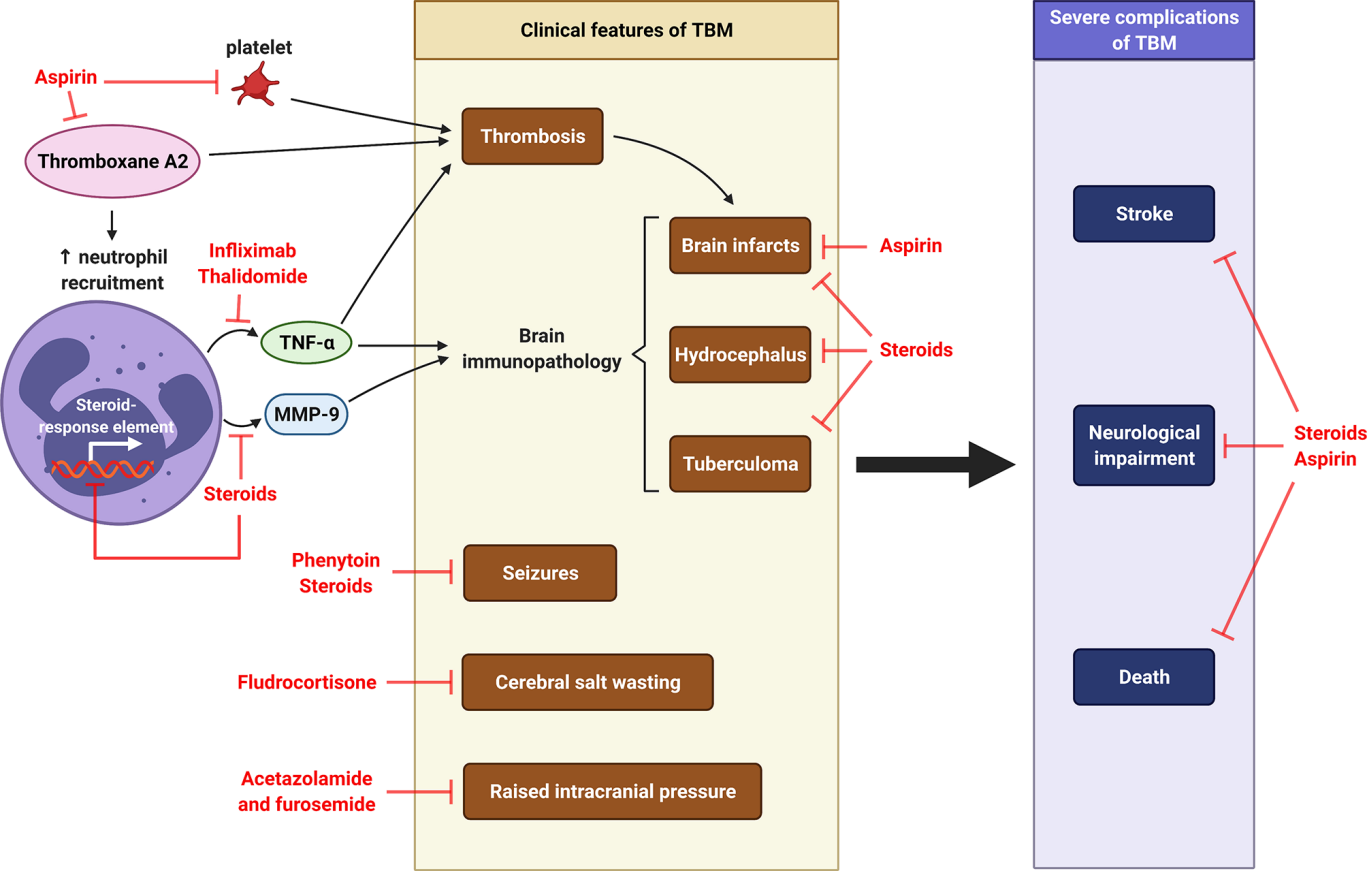
Host-directed therapy in CNS-TB. Pro-coagulant thromboxane A2 causes thrombosis and subsequent formation of brain infarcts. It also increases the recruitment of neutrophils, which releases TNF-α and MMP-9, two major mediators contributing to brain immunopathology of TBM, including brain infarcts, hydrocephalus and tuberculoma. Other clinical features of TBM are cerebral salt wasting, seizures and raised intracranial pressure. Severe complications of TBM are stroke, neurological impairment and death. Aspirin is used to inhibit thromboxane A2, prevent brain infarcts and reduce stroke and mortality (245, 246). Thalidomide is TNF-α antagonist functions to reduce thrombosis and brain immunopathology in TBM (11). Corticosteroid dexamethasone can inhibit MMP-9 secretion and further reduce the consequent brain immunopathology (10, 247), while fludrocortisone can improve cerebral salt wasting (248). The use of steroid may therefore reduce neurological impairment and death. The anti-seizure phenytoin is taken to control seizures in TBM, while the diuretics acetazolamide and furosemide can be used to manage raised intracranial pressure in TBM (249). Illustration created with Biorender.com.

As the infection progresses, TB granulomas evolve from a solid, non-necrotic structure that is capable of controlling *M. tb* growth to a necrotic phenotype that leads to host tissue damage and bacterial persistence (244). Host-directed therapies (HDTs) are adjunctive treatment with ATT to mitigate the TB immunopathology by reducing inflammation and reprogramming granuloma structure (244). Treatment that ameliorate the neutrophil-driven inflammatory responses should be considered. To date, the three major HDTs being investigated for CNS-TB management are steroids, aspirin and anti-TNF-α agents (**Figure 8**). Clinical features of TBM such as seizure and raised intracranial pressure may require drugs such as anti-epileptics, diuretics acetazolamide and furosemide (249).

### MMP Inhibition as Potential Developments in the Field of Therapeutics

Given the pivotal role of MMPs in BBB breakdown and CNS tissue destruction in CNS-TB, there is increasing evidence that targeting MMPs or their upstream regulatory pathways may improve treatment outcomes (250). Glucocorticoids such as dexamethasone are established adjunctive therapies for CNS-TB, and adjunct dexamethasone reduces mortality of TBM patients by 30% (62). However, the mechanism for this effect is not understood. *In vitro* CNS-TB studies have demonstrated dexamethasone suppressed mRNA expression and secretion of MMP-1 and -3, but not TIMP-1 and -2 (176). This suggests that dexamethasone may tip the protease: anti-protease balance in favor of reduced overall proteolytic activity, thereby reversing the matrix degrading phenotype in CNS-TB, and may be the mechanism by which corticosteroids improve outcome in TBM patients. The specific mode of action of dexamethasone on MMP expression is corroborated by a clinical study where adjunctive dexamethasone significantly reduced CSF MMP-9 concentrations in TBM patients, but had no effect on other MMPs, TIMPs, cytokines or chemokines (10, 247). In addition, CSF MMP-9 concentrations showed a strong correlation with CSF neutrophil count, indicating a central role of neutrophils in TBM pathogenesis (10). However, *in vitro* CNS-TB studies found anti-TNF-α treatment, but not dexamethasone, suppressed neutrophil MMP-9 secretion (9). The authors postulated that other MMP-9-secreting CNS cells such as astrocytes, microglia and neurons may contribute to the total suppression of CSF MMP-9 observed in the adjunctive dexamethasone-treated TBM patients.

Although MMP secretion may be affected by medications, drugs that specifically target MMPs, in particular MMP-9, would minimize off-target effects such as immunosuppression as in the use of steroids. To date, there is only one study that investigated the effect of specific MMP inhibition in an animal model of CNS-TB. Adjunctive SB-3CT, which is an inhibitor specific for MMP-2 and -9, appeared more effective than dexamethasone in *M. tb* clearance and MMP-9 suppression in a murine CNS-TB model (251). Nonetheless, further studies are needed to determine the role of MMP inhibition in improving CNS-TB treatment outcomes.

### Steroids and Controversies on Its Use to Decrease Neurological Sequelae

Corticosteroids, which include prednisolone, methylprednisolone and dexamethasone, are anti-inflammatory drugs commonly used to treat autoimmune diseases. They are known to decrease the secretion of inflammatory mediators from neutrophils. The use of corticosteroids as an adjunctive HDT for TBM is thought to reduce mortality by decreasing inflammation in the brain and its associated blood vessels, as well as reducing intracranial pressure (252). However, corticosteroids can suppress the host immune system, resulting in uncontrolled *M. tb* growth and reduced meningeal inflammation, which then reduces the ability of therapeutic drugs to cross the BBB (252). Thus, the benefit of corticosteroids as an adjunct therapy for TBM remains controversial. A review of 1,337 participants across 9 clinical trials concluded that adjunctive corticosteroids improved the short-term survival of TBM patients by approximately 25%, but were ineffective in mitigating against neurological disabilities (252). However, serial brain MRI conducted on dexamethasone-treated TBM patients suggested a reduced incidence rate of hydrocephalus and infarction (253). An immunological study that evaluated the effect of adjunctive dexamethasone on TBM treatment reported prolonged inflammatory responses in all TBM patients regardless of treatment group (247). Dexamethasone slightly decreased CSF IFN-γ concentrations, but did not alter immunological and routine biochemical indices of inflammation or peripheral blood monocyte and T cell responses to *M. tb* antigens (254). This indicates that the improved survival in dexamethasone-treated TBM patients was not due to an attenuation of inflammatory mediators in the CSF or a suppression of peripheral immune responses to *M. tb* antigens (254). The mechanism of action of adjunctive corticosteroids in reducing mortality is uncertain but adjunctive dexamethasone significantly suppressed CSF MMP-9 concentrations, which may be neutrophil-derived, in TBM patients (10, 247).

However, the utility of adjunctive corticosteroids in reducing neurological complications associated with CNS-TB remains controversial. A recent study on patients with CNS tuberculomas reported that an intensified adjunctive dexamethasone therapy (for several months up to 18 months) led to an improvement of neurological symptoms such as seizures, stupor and disturbed vision and a complete resolution of CNS lesions (255). The authors demonstrated that when dexamethasone was tapered according to the guidelines for TBM, all patients exhibited neurological deterioration which immediately improved upon increasing the dexamethasone dose (255). This discrepancy may be attributed to the possibility that most CNS-TB studies and treatment regime do not discriminate between the different entities of CNS-TB such as TBM and intracranial tuberculomas (255). The effect of adjunctive corticosteroid treatment in CNS-TB had been evaluated only for TBM patients (252), but not for patients with intracranial tuberculomas without meningitis. This study, together with a few others, that showed worsening of symptoms coinciding with the reduction or termination of corticosteroids (256–258), strongly suggests that the duration and dosage of adjunctive corticosteroid therapy in CNS tuberculomas are different from the TBM regime. It also highlights the importance of future CNS-TB clinical studies to clearly distinguish between TBM and CNS tuberculomas so that they can be more effectively managed.

### Aspirin

Even with effective ATT, up to 40% of surviving CNS-TB patients sustain neurological morbidities due to strokes (245). This led to the use of adjunctive aspirin for the prevention of ischemic strokes in TBM treatment (245, 246, 259). In the first randomized controlled trial of 118 Indian adult TBM patients, aspirin was associated with a non-significant reduction in stroke at 3 months, and a significant reduction in mortality (246). The second randomized controlled trial compared low (75 mg/kg)- and high (100 mg/kg)-dose aspirin and placebo in 146 South African pediatric TBM patients (259). Although adjunctive aspirin (regardless of dosage) showed no effect on mortality and morbidity, the outcome in high-dose aspirin group compared favorably with other treatment groups despite a significantly younger age and more severe neurological co-morbidity, which warrants further investigation of aspirin in TBM (259). While low-dose aspirin (75-150 mg) is sufficient to prevent ischemic cerebrovascular disease, higher-dose aspirin (>600 mg) is required for its anti-inflammatory effects (260, 261). In addition, aspirin’s inhibitory effect on platelets and thrombus formation may explain its role in reducing stroke-related mortality (262). A trial which compared low (81 mg)- and high (1000 mg)-dose aspirin and placebo in 120 adult Vietnamese TBM patients found aspirin to inhibit in a dose-dependent manner on pro-coagulant thromboxane A2 and upregulation of CSF protectins that promote resolution of inflammation (245). This finding supports aspirin in preventing TBM-related brain infarction by its anti-thrombotic, anti-inflammatory, and pro-resolving properties, and a larger study is needed to confirm the beneficial role of aspirin in preventing strokes in TBM patients (245).

Aspirin treatment likely exert its therapeutic effects by resolving neutrophil-mediated inflammation. In murine experiments, aspirin-induced anti-inflammatory lipoxins production to inhibit neutrophil- and platelet-mediated lung inflammation (263) and decreased systemic neutrophilic recruitment in pulmonary TB (264). Aspirin has also been shown to reduce NETs formation in PMA-stimulated human neutrophils (265). In a human model of acute respiratory distress syndrome, aspirin reduced pulmonary neutrophilia and MMP-8, -9 (266). However, the effects of aspirin on the neutrophil-derived MMP-8, -9 in CNS-TB have not been studied.

### Anti-TNF-α and Other Approaches to Host-Directed Therapies

Adjunctive therapy with thalidomide, a TNF-α antagonist, has demonstrated an improvement in survival and neurological outcome in rabbits, but not in human CNS-TB (11). Not only do human studies failed to show a correlation between TNF-α levels and disease severity or outcome, a clinical trial of adjunctive thalidomide in paediatric TBM patients was prematurely terminated due to adverse events and a lack of benefit in the thalidomide arm (11).

In summary, existing trials on the use of steroids as adjunctive therapy for CNS-TB showed that they may be beneficial in improving the survival of CNS-TB patients by reducing CSF MMP-9 concentrations, thereby reducing brain immunopathology such as infarcts and hydrocephalus. Conversely, adjunct aspirin and anti-TNF-α may mitigate CNS-TB-associated neurological disabilities and death by reducing stroke occurrence. The use of other host-directed adjunctive therapy in CNS-TB, including MMP inhibition and inhibiting other neutrophil mediators, remains to be evaluated to further improve mortality and neurological outcomes.

## Conclusion

Neutrophils play a critical role in driving CNS-TB immunopathology, with substantial evidence highlighting neutrophil-mediators in the cerebral inflammation, tissue destruction and thrombosis. Neutrophil antimicrobial arsenal such as NETs, serine proteases, MMPs and ROS are part of the innate immune response in CNS-TB, while neutrophils also cross-talk with other immune cell types, to contain *M. tb* infection. However, neutrophil-mediated immune responses may also drive CNS-TB immunopathology. While the research of adjunctive therapies in CNS-TB using steroids, aspirin, and anti-TNF-α show promise, CNS-TB patients continue to develop severe neurological morbidity despite treatment. Given this limitation, a better understanding of the mechanisms underlying CNS-TB immunopathology in the context of neutrophils and MMPs may well lead to more effective host-directed treatments.

## Author Contributions

CO and XYP conceived the review. XYP and FL wrote the first draft. XYP, FL, JF, and CO revised and re-drafted the article for critical intellectual content and approved the submitted version.

## Funding

CO is supported by NMRC/TA/0042/2015, CSAINV17nov014 and National University Health System (NUHS/RO/2017/092/SU/01). XP is supported by a postgraduate scholarship from the Yong Loo Lin School of Medicine, National University of Singapore. JF acknowledges funding to his group from the Medical Research Council (UK), Rosetrees Trust and The Wellcome Trust for work described in the review.

## Conflict of Interest

CO received speaking fees from Qiagen outside this work. The authors declare that the research was conducted in the absence of any commercial or financial relationships that could be construed as a potential conflict of interest.

## Publisher’s Note

All claims expressed in this article are solely those of the authors and do not necessarily represent those of their affiliated organizations, or those of the publisher, the editors and the reviewers. Any product that may be evaluated in this article, or claim that may be made by its manufacturer, is not guaranteed or endorsed by the publisher.

## References

[1] Global Tuberculosis Report 2020. Geneva: World Health Organization. (2020). p. 232. Licence: CC BY-NC-SA 3.0 IGO

[2] Cherian A Thomas SV. Central Nervous System Tuberculosis. Afr Health Sci (2011) 11(1):116–27. PMC3092316 21572867

[3] Garg RK. Tuberculosis of the Central Nervous System. Postgrad Med J (1999) 75(881):133–40. doi: 10.1136/pgmj.75.881.133 PMC1741157 10448488

[4] Leonard JM. Central Nervous System Tuberculosis. Microbiol Spectr (2017) 5(2). doi: 10.1128/microbiolspec.TNMI7-0044-2017 PMC11687486 28281443

[5] Rom WN Garay SM. Tuberculosis. 2 Ed. Philadelphia: Lippincott Williams & Wilkins (2004).

[6] Dallenga T Schaible UE. Neutrophils in Tuberculosis–First Line of Defence or Booster of Disease and Targets for Host-Directed Therapy? Pathog Dis (2016) 74(3). doi: 10.1093/femspd/ftw012 26903072

[7] Ong CW Elkington PT Brilha S Ugarte-Gil C Tome-Esteban MT Tezera LB. Neutrophil-Derived MMP-8 Drives AMPK-Dependent Matrix Destruction in Human Pulmonary Tuberculosis. PLoS Pathog (2015) 11(5):e1004917. doi: 10.1371/journal.ppat.1004917 25996154 PMC4440706

[8] Nwongbouwoh Muefong C Owolabi O Donkor S Charalambous S Bakuli A Rachow A. Neutrophils Contribute to Severity of Tuberculosis Pathology and Recovery From Lung Damage Pre- and Post-Treatment. Clin Infect Dis (2021). doi: 10.1093/cid/ciab729 PMC9155606 34427644

[9] Ong CW Pabisiak PJ Brilha S Singh P Roncaroli F Elkington PT. Complex Regulation of Neutrophil-Derived MMP-9 Secretion in Central Nervous System Tuberculosis. J Neuroinflamm (2017) 14(1):31. doi: 10.1186/s12974-017-0801-1 PMC5294728 28173836

[10] Green JA Tran CT Farrar JJ Nguyen MT Nguyen PH Dinh SX. Dexamethasone, Cerebrospinal Fluid Matrix Metalloproteinase Concentrations and Clinical Outcomes in Tuberculous Meningitis. PLoS One (2009) 4(9):e7277. doi: 10.1371/journal.pone.0007277 19789647 PMC2748711

[11] Tsenova L Mangaliso B Muller G Chen Y Freedman VH Stirling D. Use of IMiD3, a Thalidomide Analog, as an Adjunct to Therapy for Experimental Tuberculous Meningitis. Antimicrob Agents Chemother (2002) 46(6):1887–95. doi: 10.1128/AAC.46.6.1887-1895.2002 PMC127267 12019105

[12] Tsenova L Sokol K Freedman VH Kaplan G. A Combination of Thalidomide Plus Antibiotics Protects Rabbits From Mycobacterial Meningitis-Associated Death. J Infect Dis (1998) 177(6):1563–72. doi: 10.1086/515327 9607834

[13] Tsenova L Bergtold A Freedman VH Young RA Kaplan G. Tumor Necrosis Factor Alpha is a Determinant of Pathogenesis and Disease Progression in Mycobacterial Infection in the Central Nervous System. Proc Natl Acad Sci USA (1999) 96(10):5657–62. doi: 10.1073/pnas.96.10.5657 PMC21916 10318940

[14] Braian C Hogea V Stendahl O. Mycobacterium Tuberculosis- Induced Neutrophil Extracellular Traps Activate Human Macrophages. J Innate Immun (2013) 5(6):591–602. doi: 10.1159/000348676 23635526 PMC6741595

[15] de Bont CM Boelens WC Pruijn GJM. NETosis, Complement, and Coagulation: A Triangular Relationship. Cell Mol Immunol (2019) 16(1):19–27. doi: 10.1038/s41423-018-0024-0 29572545 PMC6318284

[16] Ducroux C Di Meglio L Loyau S Delbosc S Boisseau W Deschildre C. Thrombus Neutrophil Extracellular Traps Content Impair tPA-Induced Thrombolysis in Acute Ischemic Stroke. Stroke (2018) 49(3):754–7. doi: 10.1161/STROKEAHA.117.019896 29438080

[17] Peto HM Pratt RH Harrington TA LoBue PA Armstrong LR. Epidemiology of Extrapulmonary Tuberculosis in the United States, 1993-2006. Clin Infect Dis (2009) 49(9):1350–7. doi: 10.1086/605559 19793000

[18] Wilkinson RJ Rohlwink U Misra UK van Crevel R Mai NTH Dooley KE. Tuberculous Meningitis. Nat Rev Neurol (2017) 13(10):581–98. doi: 10.1038/nrneurol.2017.120 28884751

[19] Nguyen DT Agarwal S Graviss EA. Trends of Tuberculosis Meningitis and Associated Mortality in Texas, 2010-2017, a Large Population-Based Analysis. PLoS One (2019) 14(2):e0212729. doi: 10.1371/journal.pone.0212729 30817805 PMC6395025

[20] UK Health Security Agency. Tuberculosis in England: 2020. London: UK Health Security Agency (2021).

[21] Souza CH Yamane A Pandini JC Ceretta LB Ferraz F da Luz GD. Incidence of Tuberculous Meningitis in the State of Santa Catarina, Brazil. Rev Soc Bras Med Trop (2014) 47(4):483–9. doi: 10.1590/0037-8682-0122-2014 25229290

[22] Ducomble T Tolksdorf K Karagiannis I Hauer B Brodhun B Haas W. The Burden of Extrapulmonary and Meningitis Tuberculosis: An Investigation of National Surveillance Data, Germany, 2002 to 2009. Euro Surveill (2013) 18(12). doi: 10.2807/ese.18.12.20436-en 23557944

[23] Phypers M Harris T Power C. CNS Tuberculosis: A Longitudinal Analysis of Epidemiological and Clinical Features. Int J Tuberc Lung Dis (2006) 10(1):99–103. 16466045

[24] Rieder HL Snider DE Jr Cauthen GM. Extrapulmonary Tuberculosis in the United States. Am Rev Respir Dis (1990) 141(2):347–51. doi: 10.1164/ajrccm/141.2.347 2301852

[25] Farer LS Lowell AM Meador MP. Extrapulmonary Tuberculosis in the United States. Am J Epidemiol (1979) 109(2):205–17. doi: 10.1093/oxfordjournals.aje.a112675 425959

[26] Berenguer J Moreno S Laguna F Vicente T Adrados M Ortega A. Tuberculous Meningitis in Patients Infected With the Human Immunodeficiency Virus. N Engl J Med (1992) 326(10):668–72. doi: 10.1056/NEJM199203053261004 1346547

[27] Dube MP Holtom PD Larsen RA. Tuberculous Meningitis in Patients With and Without Human Immunodeficiency Virus Infection. Am J Med (1992) 93(5):520–4. doi: 10.1016/0002-9343(92)90579-Z 1442854

[28] Rana FS Hawken MP Mwachari C Bhatt SM Abdullah F Ng'ang'a LW. Autopsy Study of HIV-1-Positive and HIV-1-Negative Adult Medical Patients in Nairobi, Kenya. J Acquir Immune Defic Syndr (2000) 24(1):23–9. doi: 10.1097/00042560-200005010-00004 10877491

[29] Schaller MA Wicke F Foerch C Weidauer S. Central Nervous System Tuberculosis : Etiology, Clinical Manifestations and Neuroradiological Features. Clin Neuroradiol (2019) 29(1):3–18. doi: 10.1007/s00062-018-0726-9 30225516

[30] Daniele B. Characteristics of Central Nervous System Tuberculosis in a Low-Incidence Country: A Series of 20 Cases and a Review of the Literature. Jpn J Infect Dis (2014) 67(1):50–3. doi: 10.7883/yoken.67.50 24451103

[31] Soria J Metcalf T Mori N Newby RE Montano SM Huaroto L. Mortality in Hospitalized Patients With Tuberculous Meningitis. BMC Infect Dis (2019) 19(1):9. doi: 10.1186/s12879-018-3633-4 30611205 PMC6321688

[32] Seddon JA Shingadia D. Epidemiology and Disease Burden of Tuberculosis in Children: A Global Perspective. Infect Drug Resist (2014) 7:153–65. doi: 10.2147/IDR.S45090 PMC4069045 24971023

[33] Miftode EG Dorneanu OS Leca DA Juganariu G Teodor A Hurmuzache M. Tuberculous Meningitis in Children and Adults: A 10-Year Retrospective Comparative Analysis. PLoS One (2015) 10(7):e0133477. doi: 10.1371/journal.pone.0133477 26186004 PMC4506084

[34] El Sahly HM Teeter LD Pan X Musser JM Graviss EA. Mortality Associated With Central Nervous System Tuberculosis. J Infect (2007) 55(6):502–9. doi: 10.1016/j.jinf.2007.08.008 PMC2174908 17920686

[35] Jaipuriar RS Garg RK Rizvi I Malhotra HS Kumar N Jain A. Early Mortality Among Immunocompetent Patients of Tuberculous Meningitis: A Prospective Study. Am J Trop Med Hyg (2019) 101(2):357–61. doi: 10.4269/ajtmh.19-0098 PMC6685555 31237232

[36] Tsai KS Chang HL Chien ST Chen KL Chen KH Mai MH. Childhood Tuberculosis: Epidemiology, Diagnosis, Treatment, and Vaccination. Pediatr Neonatol (2013) 54(5):295–302. doi: 10.1016/j.pedneo.2013.01.019 23597517

[37] Qian X Nguyen DT Lyu J Albers AE Bi X Graviss EA. Risk Factors for Extrapulmonary Dissemination of Tuberculosis and Associated Mortality During Treatment for Extrapulmonary Tuberculosis. Emerg Microbes Infect (2018) 7(1):102. doi: 10.1038/s41426-018-0106-1 29872046 PMC5988830

[38] Ong CW Elkington PT Friedland JS. Tuberculosis, Pulmonary Cavitation, and Matrix Metalloproteinases. Am J Respir Crit Care Med (2014) 190(1):9–18. doi: 10.1164/rccm.201311-2106PP 24713029 PMC4226026

[39] Rock RB Olin M Baker CA Molitor TW Peterson PK. Central Nervous System Tuberculosis: Pathogenesis and Clinical Aspects. Clin Microbiol Rev (2008) 21(2):243–61. doi: 10.1128/CMR.00042-07 PMC2292571 18400795

[40] Moule MG Cirillo JD. Mycobacterium Tuberculosis Dissemination Plays a Critical Role in Pathogenesis. Front Cell Infect Microbiol (2020) 10:65. doi: 10.3389/fcimb.2020.00065 32161724 PMC7053427

[41] Davis AG Rohlwink UK Proust A Figaji AA Wilkinson RJ. The Pathogenesis of Tuberculous Meningitis. J Leukoc Biol (2019) 105(2):267–80. doi: 10.1002/JLB.MR0318-102R PMC6355360 30645042

[42] Rich A. The Pathogenesis of Tuberculous Meningitis. Bull Johns Hopkins Hosp (1933) 52:5–37.

[43] Blacklock JWSaMAG. Tuberculosis Meningitis: Problems in Pathogenesis and Treatment. J Pathol Bacteriol (1935) 40:489–502. doi: 10.1002/path.1700400308

[44] Donald PR Schaaf HS Schoeman JF. Tuberculous Meningitis and Miliary Tuberculosis: The Rich Focus Revisited. J Infect (2005) 50(3):193–5. doi: 10.1016/j.jinf.2004.02.010 15780412

[45] Macgregor AR Green CA. Tuberculosis of the Central Nervous System, With Special Reference to Tuberculous Meningitis. J Pathol Bacteriol (1937) 45:613–45. doi: 10.1002/path.1700450312

[46] Horne NW. Tuberculous Meningitis: Problems in Pathogenesis and Treatment. Edinb Med J (1951) 58(9):413–29. PMC5288073 14872801

[47] Zaharie SD Franken DJ van der Kuip M van Elsland S de Bakker BS Hagoort J. The Immunological Architecture of Granulomatous Inflammation in Central Nervous System Tuberculosis. Tuberculosis (Edinb) (2020) 125:102016. doi: 10.1016/j.tube.2020.102016 33137697

[48] Arvanitakis Z Long RL Hershfield ES Manfreda J Kabani A Kunimoto D. M. Tuberculosis Molecular Variation in CNS Infection: Evidence for Strain-Dependent Neurovirulence. Neurology (1998) 50(6):1827–32. doi: 10.1212/wnl.50.6.1827 9633735

[49] Bidstrup C Andersen PH Skinhoj P Andersen AB. Tuberculous Meningitis in a Country With a Low Incidence of Tuberculosis: Still a Serious Disease and a Diagnostic Challenge. Scand J Infect Dis (2002) 34(11):811–4. doi: 10.1080/0036554021000026938 12578148

[50] Thwaites GE van Toorn R Schoeman J. Tuberculous Meningitis: More Questions, Still Too Few Answers. Lancet Neurol (2013) 12(10):999–1010. doi: 10.1016/S1474-4422(13)70168-6 23972913

[51] Hopewell PC. A Clinical View of Tuberculosis. Radiol Clin North Am (1995) 33(4):641–53. 7610236

[52] al-Deeb SM Yaqub BA Sharif HS Motaery KR. Neurotuberculosis: A Review. Clin Neurol Neurosurg (1992) 94(Suppl):S30–3. doi: 10.1016/0303-8467(92)90014-T 1320510

[53] Garg RK Malhotra HS Jain A. Neuroimaging in Tuberculous Meningitis. Neurol India (2016) 64(2):219–27. doi: 10.4103/0028-3886.177608 26954796

[54] Bernaerts A Vanhoenacker FM Parizel PM Van Goethem JW Van Altena R Laridon A. Tuberculosis of the Central Nervous System: Overview of Neuroradiological Findings. Eur Radiol (2003) 13(8):1876–90. doi: 10.1007/s00330-002-1608-7 12942288

[55] Andronikou S Smith B Hatherhill M Douis H Wilmshurst J. Definitive Neuroradiological Diagnostic Features of Tuberculous Meningitis in Children. Pediatr Radiol (2004) 34(11):876–85. doi: 10.1007/s00247-004-1237-1 15378213

[56] Botha H Ackerman C Candy S Carr JA Griffith-Richards S Bateman KJ. Reliability and Diagnostic Performance of CT Imaging Criteria in the Diagnosis of Tuberculous Meningitis. PLoS One (2012) 7(6):e38982. doi: 10.1371/journal.pone.0038982 22768055 PMC3387202

[57] Raut T Garg RK Jain A Verma R Singh MK Malhotra HS. Hydrocephalus in Tuberculous Meningitis: Incidence, its Predictive Factors and Impact on the Prognosis. J Infect (2013) 66(4):330–7. doi: 10.1016/j.jinf.2012.12.009 23291048

[58] Lammie GA Hewlett RH Schoeman JF Donald PR. Tuberculous Cerebrovascular Disease: A Review. J Infect (2009) 59(3):156–66. doi: 10.1016/j.jinf.2009.07.012 19635500

[59] Donald PR Schoeman JF. Tuberculous Meningitis. N Engl J Med (2004) 351(17):1719–20. doi: 10.1056/NEJMp048227 15496620

[60] Kirwan DE Chong DLW Friedland JS. Platelet Activation and the Immune Response to Tuberculosis. Front Immunol (2021) 12:631696. doi: 10.3389/fimmu.2021.631696 34093524 PMC8170316

[61] Girgis NI Sultan Y Farid Z Mansour MM Erian MW Hanna LS. Tuberculosis Meningitis, Abbassia Fever Hospital-Naval Medical Research Unit No. 3-Cairo, Egypt, From 1976 to 1996. Am J Trop Med Hyg (1998) 58(1):28–34. doi: 10.4269/ajtmh.1998.58.28 9452288

[62] Thwaites GE Nguyen DB Nguyen HD Hoang TQ Do TT Nguyen TC. Dexamethasone for the Treatment of Tuberculous Meningitis in Adolescents and Adults. N Engl J Med (2004) 351(17):1741–51. doi: 10.1056/NEJMoa040573 15496623

[63] Katti MK. Pathogenesis, Diagnosis, Treatment, and Outcome Aspects of Cerebral Tuberculosis. Med Sci Monit (2004) 10(9):RA215–29. 15328498

[64] Thwaites GE Tran TH. Tuberculous Meningitis: Many Questions, Too Few Answers. Lancet Neurol (2005) 4(3):160–70. doi: 10.1016/S1474-4422(05)70019-3 15721826

[65] Medical Research Council, Streptomycin in Tuberculosis Trials Committee. Streptomycin Treatment of Tuberculous Meningitis. Lancet (1948) 1948(6503):582–96. 18911226

[66] Jacobs RF Sunakorn P Chotpitayasunonah T Pope S Kelleher K. Intensive Short Course Chemotherapy for Tuberculous Meningitis. Pediatr Infect Dis J (1992) 11(3):194–8. doi: 10.1097/00006454-199203000-00004 1565533

[67] Kim TK Chang KH Kim CJ Goo JM Kook MC Han MH. Intracranial Tuberculoma: Comparison of MR With Pathologic Findings. AJNR Am J Neuroradiol (1995) 16(9):1903–8. PMC8338232 8693993

[68] Wasay M Kheleani BA Moolani MK Zaheer J Pui M Hasan S. Brain CT and MRI Findings in 100 Consecutive Patients With Intracranial Tuberculoma. J Neuroimaging (2003) 13(3):240–7. doi: 10.1111/j.1552-6569.2003.tb00185.x 12889171

[69] DeLance AR Safaee M Oh MC Clark AJ Kaur G Sun MZ. Tuberculoma of the Central Nervous System. J Clin Neurosci (2013) 20(10):1333–41. doi: 10.1016/j.jocn.2013.01.008 23768968

[70] Chakraborti S Mahadevan A Govindan A Nagarathna S Santosh V Yasha TC. Clinicopathological Study of Tuberculous Brain Abscess. Pathol Res Pract (2009) 205(12):815–22. doi: 10.1016/j.prp.2009.05.012 19608350

[71] Kumar R Pandey CK Bose N Sahay S. Tuberculous Brain Abscess: Clinical Presentation, Pathophysiology and Treatment (in Children). Childs Nerv Syst (2002) 18(3-4):118–23. doi: 10.1007/s00381-002-0575-2 11981617

[72] Whitener DR. Tuberculous Brain Abscess. Report of a Case and Review of the Literature. Arch Neurol (1978) 35(3):148–55. doi: 10.1001/archneur.1978.00500270030007 629659

[73] de Vries HE Kuiper J de Boer AG Van Berkel TJ Breimer DD. The Blood-Brain Barrier in Neuroinflammatory Diseases. Pharmacol Rev (1997) 49(2):143–55. 9228664

[74] Nguyen L Pieters J. The Trojan Horse: Survival Tactics of Pathogenic Mycobacteria in Macrophages. Trends Cell Biol (2005) 15(5):269–76. doi: 10.1016/j.tcb.2005.03.009 15866031

[75] Be NA Kim KS Bishai WR Jain SK. Pathogenesis of Central Nervous System Tuberculosis. Curr Mol Med (2009) 9(2):94–9. doi: 10.2174/156652409787581655 PMC4486069 19275620

[76] Jain SK Paul-Satyaseela M Lamichhane G Kim KS Bishai WR. Mycobacterium Tuberculosis Invasion and Traversal Across an *In Vitro* Human Blood-Brain Barrier as a Pathogenic Mechanism for Central Nervous System Tuberculosis. J Infect Dis (2006) 193(9):1287–95. doi: 10.1086/502631 16586367

[77] Be NA Bishai WR Jain SK. Role of Mycobacterium Tuberculosis pknD in the Pathogenesis of Central Nervous System Tuberculosis. BMC Microbiol (2012) 12:7. doi: 10.1186/1471-2180-12-7 22243650 PMC3322341

[78] Brilha S Ong CWM Weksler B Romero N Couraud PO Friedland JS. Matrix Metalloproteinase-9 Activity and a Downregulated Hedgehog Pathway Impair Blood-Brain Barrier Function in an *In Vitro* Model of CNS Tuberculosis. Sci Rep (2017) 7(1):16031. doi: 10.1038/s41598-017-16250-3 29167512 PMC5700087

[79] Francisco NM Hsu NJ Keeton R Randall P Sebesho B Allie N. TNF-Dependent Regulation and Activation of Innate Immune Cells are Essential for Host Protection Against Cerebral Tuberculosis. J Neuroinflamm (2015) 12:125. doi: 10.1186/s12974-015-0345-1 PMC4488051 26112704

[80] Ong CWM Fox K Ettorre A Elkington PT Friedland JS. Hypoxia Increases Neutrophil-Driven Matrix Destruction After Exposure to Mycobacterium Tuberculosis. Sci Rep (2018) 8(1):11475. doi: 10.1038/s41598-018-29659-1 30065292 PMC6068197

[81] Sawant KV McMurray DN. Guinea Pig Neutrophils Infected With Mycobacterium Tuberculosis Produce Cytokines Which Activate Alveolar Macrophages in Noncontact Cultures. Infect Immun (2007) 75(4):1870–7. doi: 10.1128/IAI.00858-06 PMC1865707 17283104

[82] Hilda JN Das SD. TLR Stimulation of Human Neutrophils Lead to Increased Release of MCP-1, MIP-1alpha, IL-1beta, IL-8 and TNF During Tuberculosis. Hum Immunol (2016) 77(1):63–7. doi: 10.1016/j.humimm.2015.10.005 26472013

[83] Hilda JN Narasimhan M Das SD. Neutrophils From Pulmonary Tuberculosis Patients Show Augmented Levels of Chemokines MIP-1alpha, IL-8 and MCP-1 Which Further Increase Upon *In Vitro* Infection With Mycobacterial Strains. Hum Immunol (2014) 75(8):914–22. doi: 10.1016/j.humimm.2014.06.020 24994463

[84] Cannas S Molicotti P Bua A Usai D Sechi LA Scanu AM. Interaction Between Mycobacterium Tuberculosis, Mycobacterium Bovis, Mycobacterium Avium Subspecies Paratuberculosis With the Enteric Glia and Microglial Cells. Gut Pathog (2011) 3:19. doi: 10.1186/1757-4749-3-19 22151930 PMC3253042

[85] Fleischmann J Golde DW Weisbart RH Gasson JC. Granulocyte-Macrophage Colony-Stimulating Factor Enhances Phagocytosis of Bacteria by Human Neutrophils. Blood (1986) 68(3):708–11. doi: 10.1182/blood.V68.3.708.708 3488777

[86] Blomgran R Ernst JD. Lung Neutrophils Facilitate Activation of Naive Antigen-Specific CD4+ T Cells During Mycobacterium Tuberculosis Infection. J Immunol (2011) 186(12):7110–9. doi: 10.4049/jimmunol.1100001 PMC3376160 21555529

[87] Kang DD Lin Y Moreno JR Randall TD Khader SA. Profiling Early Lung Immune Responses in the Mouse Model of Tuberculosis. PLoS One (2011) 6(1):e16161. doi: 10.1371/journal.pone.0016161 21249199 PMC3020951

[88] Blomgran R Desvignes L Briken V Ernst JD. Mycobacterium Tuberculosis Inhibits Neutrophil Apoptosis, Leading to Delayed Activation of Naive CD4 T Cells. Cell Host Microbe (2012) 11(1):81–90. doi: 10.1016/j.chom.2011.11.012 22264515 PMC3266554

[89] Flower RJ. Prostaglandins, Bioassay and Inflammation. Br J Pharmacol (2006) 147 Suppl 1:S182–92. doi: 10.1038/sj.bjp.0706506 PMC1760729 16402103

[90] Medeiros AI Silva CL Malheiro A Maffei CM Faccioli LH. Leukotrienes are Involved in Leukocyte Recruitment Induced by Live Histoplasma Capsulatum or by the Beta-Glucan Present in Their Cell Wall. Br J Pharmacol (1999) 128(7):1529–37. doi: 10.1038/sj.bjp.0702912 PMC1571769 10602333

[91] Nourshargh S. Mechanisms of Neutrophil and Eosinophil Accumulation *In Vivo* . Am Rev Respir Dis (1993) 148(6 Pt 2):S60–4. doi: 10.1164/ajrccm/148.6_Pt_2.S60 8256924

[92] Serhan CN Chiang N Van Dyke TE. Resolving Inflammation: Dual Anti-Inflammatory and Pro-Resolution Lipid Mediators. Nat Rev Immunol (2008) 8(5):349–61. doi: 10.1038/nri2294 PMC2744593 18437155

[93] Lemos HP Grespan R Vieira SM Cunha TM Verri WA Jr Fernandes KS. Prostaglandin Mediates IL-23/IL-17-Induced Neutrophil Migration in Inflammation by Inhibiting IL-12 and IFNgamma Production. Proc Natl Acad Sci U S A (2009) 106(14):5954–9. doi: 10.1073/pnas.0812782106 PMC2667068 19289819

[94] Lammermann T. In the Eye of the Neutrophil Swarm-Navigation Signals That Bring Neutrophils Together in Inflamed and Infected Tissues. J Leukoc Biol (2016) 100(1):55–63. doi: 10.1189/jlb.1MR0915-403 26416718

[95] de Oliveira S Rosowski EE Huttenlocher A. Neutrophil Migration in Infection and Wound Repair: Going Forward in Reverse. Nat Rev Immunol (2016) 16(6):378–91. doi: 10.1038/nri.2016.49 PMC5367630 27231052

[96] Palmblad JE Lerner R. Leukotriene B4-Induced Hyperadhesiveness of Endothelial Cells for Neutrophils: Relation to CD54. Clin Exp Immunol (1992) 90(2):300–4. doi: 10.1111/j.1365-2249.1992.tb07946.x PMC1554610 1358491

[97] Vilaplana C Marzo E Tapia G Diaz J Garcia V Cardona PJ. Ibuprofen Therapy Resulted in Significantly Decreased Tissue Bacillary Loads and Increased Survival in a New Murine Experimental Model of Active Tuberculosis. J Infect Dis (2013) 208(2):199–202. doi: 10.1093/infdis/jit152 23564636

[98] Nathan C. Neutrophils and Immunity: Challenges and Opportunities. Nat Rev Immunol (2006) 6(3):173–82. doi: 10.1038/nri1785 16498448

[99] Dallenga T Repnik U Corleis B Eich J Reimer R Griffiths GW. M. Tuberculosis-Induced Necrosis of Infected Neutrophils Promotes Bacterial Growth Following Phagocytosis by Macrophages. Cell Host Microbe (2017) 22(4):519–30 e3. doi: 10.1016/j.chom.2017.09.003 29024644

[100] Corleis B Korbel D Wilson R Bylund J Chee R Schaible UE. Escape of Mycobacterium Tuberculosis From Oxidative Killing by Neutrophils. Cell Microbiol (2012) 14(7):1109–21. doi: 10.1111/j.1462-5822.2012.01783.x 22405091

[101] Dallenga T Linnemann L Paudyal B Repnik U Griffiths G Schaible UE. Targeting Neutrophils for Host-Directed Therapy to Treat Tuberculosis. Int J Med Microbiol (2018) 308(1):142–7. doi: 10.1016/j.ijmm.2017.10.001 29055689

[102] Perskvist N Long M Stendahl O Zheng L. Mycobacterium Tuberculosis Promotes Apoptosis in Human Neutrophils by Activating Caspase-3 and Altering Expression of Bax/Bcl-xL *via* an Oxygen-Dependent Pathway. J Immunol (2002) 168(12):6358–65. doi: 10.4049/jimmunol.168.12.6358 12055253

[103] Persson YA Blomgran-Julinder R Rahman S Zheng L Stendahl O. Mycobacterium Tuberculosis-Induced Apoptotic Neutrophils Trigger a Pro-Inflammatory Response in Macrophages Through Release of Heat Shock Protein 72, Acting in Synergy With the Bacteria. Microbes Infect (2008) 10(3):233–40. doi: 10.1016/j.micinf.2007.11.007 18328761

[104] Tan BH Meinken C Bastian M Bruns H Legaspi A Ochoa MT. Macrophages Acquire Neutrophil Granules for Antimicrobial Activity Against Intracellular Pathogens. J Immunol (2006) 177(3):1864–71. doi: 10.4049/jimmunol.177.3.1864 16849498

[105] Cooper AM Dalton DK Stewart TA Griffin JP Russell DG Orme IM. Disseminated Tuberculosis in Interferon Gamma Gene-Disrupted Mice. J Exp Med (1993) 178(6):2243–7. doi: 10.1084/jem.178.6.2243 PMC2191280 8245795

[106] Keane J Gershon S Wise RP Mirabile-Levens E Kasznica J Schwieterman WD. Tuberculosis Associated With Infliximab, a Tumor Necrosis Factor Alpha-Neutralizing Agent. N Engl J Med (2001) 345(15):1098–104. doi: 10.1056/NEJMoa011110 11596589

[107] Botha T Ryffel B. Reactivation of Latent Tuberculosis Infection in TNF-Deficient Mice. J Immunol (2003) 171(6):3110–8. doi: 10.4049/jimmunol.171.6.3110 12960337

[108] Ehlers S Kutsch S Ehlers EM Benini J Pfeffer K. Lethal Granuloma Disintegration in Mycobacteria-Infected TNFRp55-/- Mice is Dependent on T Cells and IL-12. J Immunol (2000) 165(1):483–92. doi: 10.4049/jimmunol.165.1.483 10861087

[109] Saunders BM Frank AA Orme IM Cooper AM. CD4 is Required for the Development of a Protective Granulomatous Response to Pulmonary Tuberculosis. Cell Immunol (2002) 216(1-2):65–72. doi: 10.1016/S0008-8749(02)00510-5 12381351

[110] Nandi B Behar SM. Regulation of Neutrophils by Interferon-Gamma Limits Lung Inflammation During Tuberculosis Infection. J Exp Med (2011) 208(11):2251–62. doi: 10.1084/jem.20110919 PMC3201199 21967766

[111] MacMicking JD Taylor GA McKinney JD. Immune Control of Tuberculosis by IFN-Gamma-Inducible LRG-47. Science (2003) 302(5645):654–9. doi: 10.1126/science.1088063 14576437

[112] Gutierrez MG Master SS Singh SB Taylor GA Colombo MI Deretic V. Autophagy is a Defense Mechanism Inhibiting BCG and Mycobacterium Tuberculosis Survival in Infected Macrophages. Cell (2004) 119(6):753–66. doi: 10.1016/j.cell.2004.11.038 15607973

[113] Mishra BB Rathinam VA Martens GW Martinot AJ Kornfeld H Fitzgerald KA. Nitric Oxide Controls the Immunopathology of Tuberculosis by Inhibiting NLRP3 Inflammasome-Dependent Processing of IL-1beta. Nat Immunol (2013) 14(1):52–60. doi: 10.1038/ni.2474 23160153 PMC3721324

[114] Brinkmann V Reichard U Goosmann C Fauler B Uhlemann Y Weiss DS. Neutrophil Extracellular Traps Kill Bacteria. Science (2004) 303(5663):1532–5. doi: 10.1126/science.1092385 15001782

[115] Ramos-Kichik V Mondragon-Flores R Mondragon-Castelan M Gonzalez-Pozos S Muniz-Hernandez S Rojas-Espinosa O. Neutrophil Extracellular Traps are Induced by Mycobacterium Tuberculosis. Tuberculosis (Edinb) (2009) 89(1):29–37. doi: 10.1016/j.tube.2008.09.009 19056316

[116] Takei H Araki A Watanabe H Ichinose A Sendo F. Rapid Killing of Human Neutrophils by the Potent Activator Phorbol 12-Myristate 13-Acetate (PMA) Accompanied by Changes Different From Typical Apoptosis or Necrosis. J Leukoc Biol (1996) 59(2):229–40. doi: 10.1002/jlb.59.2.229 8603995

[117] Jorch SK Kubes P. An Emerging Role for Neutrophil Extracellular Traps in Noninfectious Disease. Nat Med (2017) 23(3):279–87. doi: 10.1038/nm.4294 28267716

[118] Kaplan MJ Radic M. Neutrophil Extracellular Traps: Double-Edged Swords of Innate Immunity. J Immunol (2012) 189(6):2689–95. doi: 10.4049/jimmunol.1201719 PMC3439169 22956760

[119] Metzler KD Goosmann C Lubojemska A Zychlinsky A Papayannopoulos V. A Myeloperoxidase-Containing Complex Regulates Neutrophil Elastase Release and Actin Dynamics During NETosis. Cell Rep (2014) 8(3):883–96. doi: 10.1016/j.celrep.2014.06.044 PMC4471680 25066128

[120] Clark SR Ma AC Tavener SA McDonald B Goodarzi Z Kelly MM. Platelet TLR4 Activates Neutrophil Extracellular Traps to Ensnare Bacteria in Septic Blood. Nat Med (2007) 13(4):463–9. doi: 10.1038/nm1565 17384648

[121] Pilsczek FH Salina D Poon KK Fahey C Yipp BG Sibley CD. A Novel Mechanism of Rapid Nuclear Neutrophil Extracellular Trap Formation in Response to Staphylococcus Aureus. J Immunol (2010) 185(12):7413–25. doi: 10.4049/jimmunol.1000675 21098229

[122] Yipp BG Petri B Salina D Jenne CN Scott BN Zbytnuik LD. Infection-Induced NETosis is a Dynamic Process Involving Neutrophil Multitasking *In Vivo* . Nat Med (2012) 18(9):1386–93. doi: 10.1038/nm.2847 PMC4529131 22922410

[123] Rochael NC Guimaraes-Costa AB Nascimento MT DeSouza-Vieira TS Oliveira MP Garcia e Souza LF. Classical ROS-Dependent and Early/Rapid ROS-Independent Release of Neutrophil Extracellular Traps Triggered by Leishmania Parasites. Sci Rep (2015) 5:18302. doi: 10.1038/srep18302 26673780 PMC4682131

[124] Slaba I Wang J Kolaczkowska E McDonald B Lee WY Kubes P. Imaging the Dynamic Platelet-Neutrophil Response in Sterile Liver Injury and Repair in Mice. Hepatology (2015) 62(5):1593–605. doi: 10.1002/hep.28003 26202541

[125] Azzouz D Khan MA Sweezey N Palaniyar N. Two-In-One: UV Radiation Simultaneously Induces Apoptosis and NETosis. Cell Death Discov (2018) 4:51. doi: 10.1038/s41420-018-0048-3 PMC5919968 29736268

[126] Berry MP Graham CM McNab FW Xu Z Bloch SA Oni T. An Interferon-Inducible Neutrophil-Driven Blood Transcriptional Signature in Human Tuberculosis. Nature (2010) 466(7309):973–7. doi: 10.1038/nature09247 PMC3492754 20725040

[127] Moreira-Teixeira L Stimpson PJ Stavropoulos E Hadebe S Chakravarty P Ioannou M. Type I IFN Exacerbates Disease in Tuberculosis-Susceptible Mice by Inducing Neutrophil-Mediated Lung Inflammation and NETosis. Nat Commun (2020) 11(1):5566. doi: 10.1038/s41467-020-19412-6 33149141 PMC7643080

[128] Ravindran M Khan MA Palaniyar N. Neutrophil Extracellular Trap Formation: Physiology, Pathology, and Pharmacology. Biomolecules (2019) 9(8):365. doi: 10.3390/biom9080365 PMC6722781 31416173

[129] Kolaczkowska E Jenne CN Surewaard BG Thanabalasuriar A Lee WY Sanz MJ. Molecular Mechanisms of NET Formation and Degradation Revealed by Intravital Imaging in the Liver Vasculature. Nat Commun (2015) 6:6673. doi: 10.1038/ncomms7673 25809117 PMC4389265

[130] Erpenbeck L Schon MP. Neutrophil Extracellular Traps: Protagonists of Cancer Progression? Oncogene (2017) 36(18):2483–90. doi: 10.1038/onc.2016.406 27941879

[131] Khan MA D'Ovidio A Tran H Palaniyar N. Anthracyclines Suppress Both NADPH Oxidase- Dependent and -Independent NETosis in Human Neutrophils. Cancers (Basel) (2019) 11(9):1328. doi: 10.3390/cancers11091328 PMC6770146 31500300

[132] Sollberger G Choidas A Burn GL Habenberger P Di Lucrezia R Kordes S. Gasdermin D Plays a Vital Role in the Generation of Neutrophil Extracellular Traps. Sci Immunol (2018) 3(26). doi: 10.1126/sciimmunol.aar6689 30143555

[133] Guyot N Wartelle J Malleret L Todorov AA Devouassoux G Pacheco Y. Neutrophil Elastase, and Proteinase 3 Cause Severe Lung Damage and Emphysema. Am J Pathol (2014) 184(8):2197–210. doi: 10.1016/j.ajpath.2014.04.015 24929239

[134] Marais S Lai RPJ Wilkinson KA Meintjes G O'Garra A Wilkinson RJ. Inflammasome Activation Underlying Central Nervous System Deterioration in HIV-Associated Tuberculosis. J Infect Dis (2017) 215(5):677–86. doi: 10.1093/infdis/jiw561 PMC5388298 27932622

[135] Chen H Song YS Chan PH. Inhibition of NADPH Oxidase is Neuroprotective After Ischemia-Reperfusion. J Cereb Blood Flow Metab (2009) 29(7):1262–72. doi: 10.1038/jcbfm.2009.47 PMC2733333 19417757

[136] Kvietys PR Granger DN. Role of Reactive Oxygen and Nitrogen Species in the Vascular Responses to Inflammation. Free Radic Biol Med (2012) 52(3):556–92. doi: 10.1016/j.freeradbiomed.2011.11.002 PMC3348846 22154653

[137] Krizbai IA Bauer H Bresgen N Eckl PM Farkas A Szatmari E. Effect of Oxidative Stress on the Junctional Proteins of Cultured Cerebral Endothelial Cells. Cell Mol Neurobiol (2005) 25(1):129–39. doi: 10.1007/s10571-004-1378-7 PMC11529493 15962510

[138] Hilda JN Das S Tripathy SP Hanna LE. Role of Neutrophils in Tuberculosis: A Bird's Eye View. Innate Immun (2020) 26(4):240–7. doi: 10.1177/1753425919881176 PMC7251797 31735099

[139] Mastroianni CM Lancella L Mengoni F Lichtner M Santopadre P D'Agostino C. Chemokine Profiles in the Cerebrospinal Fluid (CSF) During the Course of Pyogenic and Tuberculous Meningitis. Clin Exp Immunol (1998) 114(2):210–4. doi: 10.1046/j.1365-2249.1998.00698.x PMC1905098 9822278

[140] Singh S Maniakis-Grivas G Singh UK Asher RM Mauri F Elkington PT. Interleukin-17 Regulates Matrix Metalloproteinase Activity in Human Pulmonary Tuberculosis. J Pathol (2018) 244(3):311–22. doi: 10.1002/path.5013 PMC5838784 29210073

[141] Marais S Wilkinson KA Lesosky M Coussens AK Deffur A Pepper DJ. Neutrophil-Associated Central Nervous System Inflammation in Tuberculous Meningitis Immune Reconstitution Inflammatory Syndrome. Clin Infect Dis (2014) 59(11):1638–47. doi: 10.1093/cid/ciu641 PMC4227574 25107295

[142] Lin PL Plessner HL Voitenok NN Flynn JL. Tumor Necrosis Factor and Tuberculosis. J Investig Dermatol Symp Proc (2007) 12(1):22–5. doi: 10.1038/sj.jidsymp.5650027 17502865

[143] Francisco NM Allie N Sebesho B Ryffel B Jacobs M. Complete Ablation of Tumor Necrosis Factor Decreases the Production of IgA, IgG, and IgM in Experimental Central Nervous System Tuberculosis. Iran J Basic Med Sci (2020) 23(5):680–90. doi: 10.22038/ijbms.2020.37947.9021 PMC7374998 32742607

[144] Hsu NJ Francisco NM Keeton R Allie N Quesniaux VF Ryffel B. Myeloid and T Cell-Derived TNF Protects Against Central Nervous System Tuberculosis. Front Immunol (2017) 8:180. doi: 10.3389/fimmu.2017.00180 28280495 PMC5322283

[145] Hot A Lenief V Miossec P. Combination of IL-17 and TNFalpha Induces a Pro-Inflammatory, Pro-Coagulant and Pro-Thrombotic Phenotype in Human Endothelial Cells. Ann Rheum Dis (2012) 71(5):768–76. doi: 10.1136/annrheumdis-2011-200468 22258491

[146] Parks WC Wilson CL Lopez-Boado YS. Matrix Metalloproteinases as Modulators of Inflammation and Innate Immunity. Nat Rev Immunol (2004) 4(8):617–29. doi: 10.1038/nri1418 15286728

[147] Chandler S Miller KM Clements JM Lury J Corkill D Anthony DC. Matrix Metalloproteinases, Tumor Necrosis Factor and Multiple Sclerosis: An Overview. J Neuroimmunol (1997) 72(2):155–61. doi: 10.1016/S0165-5728(96)00179-8 9042108

[148] Yang Y Estrada EY Thompson JF Liu W Rosenberg GA. Matrix Metalloproteinase-Mediated Disruption of Tight Junction Proteins in Cerebral Vessels is Reversed by Synthetic Matrix Metalloproteinase Inhibitor in Focal Ischemia in Rat. J Cereb Blood Flow Metab (2007) 27(4):697–709. doi: 10.1038/sj.jcbfm.9600375 16850029

[149] Zhang Q Zheng M Betancourt CE Liu L Sitikov A Sladojevic N. Increase in Blood-Brain Barrier (BBB) Permeability Is Regulated by MMP3 *via* the ERK Signaling Pathway. Oxid Med Cell Longev (2021) 2021:6655122. doi: 10.1155/2021/6655122 33859779 PMC8026308

[150] Kahari VM Saarialho-Kere U. Matrix Metalloproteinases in Skin. Exp Dermatol (1997) 6(5):199–213. doi: 10.1111/j.1600-0625.1997.tb00164.x 9450622

[151] Ashworth JL Murphy G Rock MJ Sherratt MJ Shapiro SD Shuttleworth CA. Fibrillin Degradation by Matrix Metalloproteinases: Implications for Connective Tissue Remodelling. Biochem J (1999) 340( Pt 1):171–81. doi: 10.1042/bj3400171 PMC1220235 10229672

[152] Rauch U. Extracellular Matrix Components Associated With Remodeling Processes in Brain. Cell Mol Life Sci (2004) 61(16):2031–45. doi: 10.1007/s00018-004-4043-x PMC11138714 15316653

[153] Sang QX Birkedal-Hansen H Van Wart HE. Proteolytic and non-Proteolytic Activation of Human Neutrophil Progelatinase B. Biochim Biophys Acta (1995) 1251(2):99–108. doi: 10.1016/0167-4838(95)00086-A 7669817

[154] Schonbeck U Mach F Libby P. Generation of Biologically Active IL-1 Beta by Matrix Metalloproteinases: A Novel Caspase-1-Independent Pathway of IL-1 Beta Processing. J Immunol (1998) 161(7):3340–6. 9759850

[155] Gurney KJ Estrada EY Rosenberg GA. Blood-Brain Barrier Disruption by Stromelysin-1 Facilitates Neutrophil Infiltration in Neuroinflammation. Neurobiol Dis (2006) 23(1):87–96. doi: 10.1016/j.nbd.2006.02.006 16624562

[156] Liu J Jin X Liu KJ Liu W. Matrix Metalloproteinase-2-Mediated Occludin Degradation and Caveolin-1-Mediated Claudin-5 Redistribution Contribute to Blood-Brain Barrier Damage in Early Ischemic Stroke Stage. J Neurosci (2012) 32(9):3044–57. doi: 10.1523/JNEUROSCI.6409-11.2012 PMC3339570 22378877

[157] Lochter A Galosy S Muschler J Freedman N Werb Z Bissell MJ. Matrix Metalloproteinase Stromelysin-1 Triggers a Cascade of Molecular Alterations That Leads to Stable Epithelial-to-Mesenchymal Conversion and a Premalignant Phenotype in Mammary Epithelial Cells. J Cell Biol (1997) 139(7):1861–72. doi: 10.1083/jcb.139.7.1861 PMC2132651 9412478

[158] McGuire JK Li Q Parks WC. Matrilysin (Matrix Metalloproteinase-7) Mediates E-Cadherin Ectodomain Shedding in Injured Lung Epithelium. Am J Pathol (2003) 162(6):1831–43. doi: 10.1016/S0002-9440(10)64318-0 PMC1868120 12759241

[159] Yu Q Stamenkovic I. Cell Surface-Localized Matrix Metalloproteinase-9 Proteolytically Activates TGF-Beta and Promotes Tumor Invasion and Angiogenesis. Genes Dev (2000) 14(2):163–76. doi: 10.1101/gad.14.2.163 PMC316345 10652271

[160] McQuibban GA Gong JH Tam EM McCulloch CA Clark-Lewis I Overall CM. Inflammation Dampened by Gelatinase A Cleavage of Monocyte Chemoattractant Protein-3. Science (2000) 289(5482):1202–6. doi: 10.1126/science.289.5482.1202 10947989

[161] Zhang K McQuibban GA Silva C Butler GS Johnston JB Holden J. HIV-Induced Metalloproteinase Processing of the Chemokine Stromal Cell Derived Factor-1 Causes Neurodegeneration. Nat Neurosci (2003) 6(10):1064–71. doi: 10.1038/nn1127 14502291

[162] Haro H Crawford HC Fingleton B Shinomiya K Spengler DM Matrisian LM. Matrix Metalloproteinase-7-Dependent Release of Tumor Necrosis Factor-Alpha in a Model of Herniated Disc Resorption. J Clin Invest (2000) 105(2):143–50. doi: 10.1172/JCI7091 PMC377426 10642592

[163] Churg A Wang RD Tai H Wang X Xie C Dai J. Macrophage Metalloelastase Mediates Acute Cigarette Smoke-Induced Inflammation *via* Tumor Necrosis Factor-Alpha Release. Am J Respir Crit Care Med (2003) 167(8):1083–9. doi: 10.1164/rccm.200212-1396OC 12522030

[164] Sternlicht MD Werb Z. How Matrix Metalloproteinases Regulate Cell Behavior. Annu Rev Cell Dev Biol (2001) 17:463–516. doi: 10.1146/annurev.cellbio.17.1.463 11687497 PMC2792593

[165] Mackay AR Hartzler JL Pelina MD Thorgeirsson UP. Studies on the Ability of 65-kDa and 92-kDa Tumor Cell Gelatinases to Degrade Type IV Collagen. J Biol Chem (1990) 265(35):21929–34. doi: 10.1016/S0021-9258(18)45827-9 2174891

[166] Brooks PC Stromblad S Sanders LC von Schalscha TL Aimes RT Stetler-Stevenson WG. Localization of Matrix Metalloproteinase MMP-2 to the Surface of Invasive Cells by Interaction With Integrin Alpha V Beta 3. Cell (1996) 85(5):683–93. doi: 10.1016/S0092-8674(00)81235-0 8646777

[167] Yu WH Woessner JF Jr. Heparan Sulfate Proteoglycans as Extracellular Docking Molecules for Matrilysin (Matrix Metalloproteinase 7). J Biol Chem (2000) 275(6):4183–91. doi: 10.1074/jbc.275.6.4183 10660581

[168] Yu WH Woessner JF Jr McNeish JD Stamenkovic I. CD44 Anchors the Assembly of Matrilysin/MMP-7 With Heparin-Binding Epidermal Growth Factor Precursor and ErbB4 and Regulates Female Reproductive Organ Remodeling. Genes Dev (2002) 16(3):307–23. doi: 10.1101/gad.925702 PMC155329 11825873

[169] Loffek S Schilling O Franzke CW. Series "Matrix Metalloproteinases in Lung Health and Disease": Biological Role of Matrix Metalloproteinases: A Critical Balance. Eur Respir J (2011) 38(1):191–208. doi: 10.1183/09031936.00146510 21177845

[170] Visse R Nagase H. Matrix Metalloproteinases and Tissue Inhibitors of Metalloproteinases: Structure, Function, and Biochemistry. Circ Res (2003) 92(8):827–39. doi: 10.1161/01.RES.0000070112.80711.3D 12730128

[171] Murphy G. Tissue Inhibitors of Metalloproteinases. Genome Biol (2011) 12(11):233. doi: 10.1186/gb-2011-12-11-233 22078297 PMC3334591

[172] Candelario-Jalil E Yang Y Rosenberg GA. Diverse Roles of Matrix Metalloproteinases and Tissue Inhibitors of Metalloproteinases in Neuroinflammation and Cerebral Ischemia. Neuroscience (2009) 158(3):983–94. doi: 10.1016/j.neuroscience.2008.06.025 PMC3584171 18621108

[173] Rosenberg GA. Matrix Metalloproteinases in Neuroinflammation. Glia (2002) 39(3):279–91. doi: 10.1002/glia.10108 12203394

[174] Tayebjee MH Nadar S Blann AD Gareth Beevers D MacFadyen RJ Lip GY. Matrix Metalloproteinase-9 and Tissue Inhibitor of Metalloproteinase-1 in Hypertension and Their Relationship to Cardiovascular Risk and Treatment: A Substudy of the Anglo-Scandinavian Cardiac Outcomes Trial (ASCOT). Am J Hypertens (2004) 17(9):764–9. doi: 10.1016/S0895-7061(04)00855-6 15363817

[175] Leppert D Leib SL Grygar C Miller KM Schaad UB Hollander GA. Matrix Metalloproteinase (MMP)-8 and MMP-9 in Cerebrospinal Fluid During Bacterial Meningitis: Association With Blood-Brain Barrier Damage and Neurological Sequelae. Clin Infect Dis (2000) 31(1):80–4. doi: 10.1086/313922 10913401

[176] Green JA Elkington PT Pennington CJ Roncaroli F Dholakia S Moores RC. Mycobacterium Tuberculosis Upregulates Microglial Matrix Metalloproteinase-1 and -3 Expression and Secretion *via* NF-kappaB- and Activator Protein-1-Dependent Monocyte Networks. J Immunol (2010) 184(11):6492–503. doi: 10.4049/jimmunol.0903811 20483790

[177] Green JA Friedland JS. Astrocyte-Leucocyte Interactions and the Mechanisms Regulating Matrix Degradation in CNS Tuberculosis. Biochem Soc Trans (2007) 35(Pt 4):686–8. doi: 10.1042/BST0350686 17635122

[178] Nuttall RK Silva C Hader W Bar-Or A Patel KD Edwards DR. Metalloproteinases are Enriched in Microglia Compared With Leukocytes and They Regulate Cytokine Levels in Activated Microglia. Glia (2007) 55(5):516–26. doi: 10.1002/glia.20478 17216595

[179] Woo MS Park JS Choi IY Kim WK Kim HS. Inhibition of MMP-3 or -9 Suppresses Lipopolysaccharide-Induced Expression of Proinflammatory Cytokines and iNOS in Microglia. J Neurochem (2008) 106(2):770–80. doi: 10.1111/j.1471-4159.2008.05430.x 18419763

[180] Sabir N Hussain T Mangi MH Zhao D Zhou X. Matrix Metalloproteinases: Expression, Regulation and Role in the Immunopathology of Tuberculosis. Cell Prolif (2019) 52(4):e12649. doi: 10.1111/cpr.12649 31199047 PMC6668971

[181] Hernandez-Pando R Orozco H Arriaga K Pavon L Rook G. Treatment With BB-94, a Broad Spectrum Inhibitor of Zinc-Dependent Metalloproteinases, Causes Deviation of the Cytokine Profile Towards Type-2 in Experimental Pulmonary Tuberculosis in Balb/c Mice. Int J Exp Pathol (2000) 81(3):199–209. doi: 10.1046/j.1365-2613.2000.00152.x 10971741 PMC2517727

[182] Izzo AA Izzo LS Kasimos J Majka S. A Matrix Metalloproteinase Inhibitor Promotes Granuloma Formation During the Early Phase of Mycobacterium Tuberculosis Pulmonary Infection. Tuberculosis (Edinb) (2004) 84(6):387–96. doi: 10.1016/j.tube.2004.07.001 15525562

[183] Taylor JL Hattle JM Dreitz SA Troudt JM Izzo LS Basaraba RJ. Role for Matrix Metalloproteinase 9 in Granuloma Formation During Pulmonary Mycobacterium Tuberculosis Infection. Infect Immun (2006) 74(11):6135–44. doi: 10.1128/IAI.02048-05 PMC1695484 16982845

[184] Harris JE Nuttall RK Elkington PT Green JA Horncastle DE Graeber MB. Monocyte-Astrocyte Networks Regulate Matrix Metalloproteinase Gene Expression and Secretion in Central Nervous System Tuberculosis *In Vitro* and *In Vivo* . J Immunol (2007) 178(2):1199–207. doi: 10.4049/jimmunol.178.2.1199 17202385

[185] Matsuura E Umehara F Hashiguchi T Fujimoto N Okada Y Osame M. Marked Increase of Matrix Metalloproteinase 9 in Cerebrospinal Fluid of Patients With Fungal or Tuberculous Meningoencephalitis. J Neurol Sci (2000) 173(1):45–52. doi: 10.1016/S0022-510X(99)00303-2 10675579

[186] Price NM Farrar J Tran TT Nguyen TH Tran TH Friedland JS. Identification of a Matrix-Degrading Phenotype in Human Tuberculosis *In Vitro* and *In Vivo* . J Immunol (2001) 166(6):4223–30. doi: 10.4049/jimmunol.166.6.4223 11238675

[187] Lee KY Kim EH Yang WS Ryu H Cho SN Lee BI. Persistent Increase of Matrix Metalloproteinases in Cerebrospinal Fluid of Tuberculous Meningitis. J Neurol Sci (2004) 220(1-2):73–8. doi: 10.1016/j.jns.2004.02.008 15140609

[188] Mailankody S Dangeti GV Soundravally R Joseph NM Mandal J Dutta TK. Cerebrospinal Fluid Matrix Metalloproteinase 9 Levels, Blood-Brain Barrier Permeability, and Treatment Outcome in Tuberculous Meningitis. PLoS One (2017) 12(7):e0181262. doi: 10.1371/journal.pone.0181262 28704492 PMC5507543

[189] Li YJ Wilkinson KA Wilkinson RJ Figaji AA Rohlwink UK. Elevated Matrix Metalloproteinase Concentrations Offer Novel Insight Into Their Role in Pediatric Tuberculous Meningitis. J Pediatr Infect Dis Soc (2020) 9(1):82–6. doi: 10.1093/jpids/piy141 30753686

[190] Majeed S Singh P Sharma N Sharma S. Title: Role of Matrix Metalloproteinase -9 in Progression of Tuberculous Meningitis: A Pilot Study in Patients at Different Stages of the Disease. BMC Infect Dis (2016) 16(1):722. doi: 10.1186/s12879-016-1953-9 27899068 PMC5129227

[191] Thwaites GE Simmons CP Than Ha Quyen N Thi Hong Chau T Phuong Mai P Thi Dung N. Pathophysiology and Prognosis in Vietnamese Adults With Tuberculous Meningitis. J Infect Dis (2003) 188(8):1105–15. doi: 10.1086/378642 14551879

[192] Rai D Garg RK Mahdi AA Jain A Verma R Tripathi AK. Cerebrospinal Fluid Cytokines and Matrix Metalloproteinases in Human Immunodeficiency Seropositive and Seronegative Patients of Tuberculous Meningitis. Ann Indian Acad Neurol (2014) 17(2):171–8. doi: 10.4103/0972-2327.132617 PMC4090842 25024567

[193] Rohlwink UK Walker NF Ordonez AA Li YJ Tucker EW Elkington PT. Matrix Metalloproteinases in Pulmonary and Central Nervous System Tuberculosis-A Review. Int J Mol Sci (2019) 20(6):1350. doi: 10.3390/ijms20061350 PMC6471445 30889803

[194] Price NM Gilman RH Uddin J Recavarren S Friedland JS. Unopposed Matrix Metalloproteinase-9 Expression in Human Tuberculous Granuloma and the Role of TNF-Alpha-Dependent Monocyte Networks. J Immunol (2003) 171(10):5579–86. doi: 10.4049/jimmunol.171.10.5579 14607966

[195] Green JA Dholakia S Janczar K Ong CW Moores R Fry J. Mycobacterium Tuberculosis-Infected Human Monocytes Down-Regulate Microglial MMP-2 Secretion in CNS Tuberculosis *via* TNFalpha, NFkappaB, P38 and Caspase 8 Dependent Pathways. J Neuroinflamm (2011) 8:46. doi: 10.1186/1742-2094-8-46 PMC3113956 21569377

[196] Brilha S Sathyamoorthy T Stuttaford LH Walker NF Wilkinson RJ Singh S. Early Secretory Antigenic Target-6 Drives Matrix Metalloproteinase-10 Gene Expression and Secretion in Tuberculosis. Am J Respir Cell Mol Biol (2017) 56(2):223–32. doi: 10.1165/rcmb.2016-0162OC PMC5359650 27654284

[197] Tadokera R Meintjes GA Wilkinson KA Skolimowska KH Walker N Friedland JS. Matrix Metalloproteinases and Tissue Damage in HIV-Tuberculosis Immune Reconstitution Inflammatory Syndrome. Eur J Immunol (2014) 44(1):127–36. doi: 10.1002/eji.201343593 PMC3992843 24136296

[198] Tai MS Viswanathan S Rahmat K Nor HM Kadir KAA Goh KJ. Cerebral Infarction Pattern in Tuberculous Meningitis. Sci Rep (2016) 6:38802. doi: 10.1038/srep38802 27958312 PMC5153843

[199] Misra UK Kalita J Maurya PK. Stroke in Tuberculous Meningitis. J Neurol Sci (2011) 303(1-2):22–30. doi: 10.1016/j.jns.2010.12.015 21272895

[200] Springer P Swanevelder S van Toorn R van Rensburg AJ Schoeman J. Cerebral Infarction and Neurodevelopmental Outcome in Childhood Tuberculous Meningitis. Eur J Paediatr Neurol (2009) 13(4):343–9. doi: 10.1016/j.ejpn.2008.07.004 18757219

[201] Zhang L Zhang X Li H Chen G Zhu M. Acute Ischemic Stroke in Young Adults With Tuberculous Meningitis. BMC Infect Dis (2019) 19(1):362. doi: 10.1186/s12879-019-4004-5 31039747 PMC6492375

[202] Chatterjee D Radotra BD Vasishta RK Sharma K. Vascular Complications of Tuberculous Meningitis: An Autopsy Study. Neurol India (2015) 63(6):926–32. doi: 10.4103/0028-3886.170086 26588628

[203] Soni N Kumar S Shimle A Ora M Bathla G Mishra P. Cerebrovascular Complications in Tuberculous Meningitis-A Magnetic Resonance Imaging Study in 90 Patients From a Tertiary Care Hospital. Neuroradiol J (2020) 33(1):3–16. doi: 10.1177/1971400919881188 31589101 PMC7005991

[204] Sharma S Goyal MK Sharma K Modi M Sharma M Khandelwal N. Cytokines do Play a Role in Pathogenesis of Tuberculous Meningitis: A Prospective Study From a Tertiary Care Center in India. J Neurol Sci (2017) 379:131–6. doi: 10.1016/j.jns.2017.06.001 28716226

[205] Rohlwink UK Mauff K Wilkinson KA Enslin N Wegoye E Wilkinson RJ. Biomarkers of Cerebral Injury and Inflammation in Pediatric Tuberculous Meningitis. Clin Infect Dis (2017) 65(8):1298–307. doi: 10.1093/cid/cix540 PMC5815568 28605426

[206] Manyelo CM Chegou NN Seddon JA Snyders CI Mutavhatsindi H Manngo PM. Serum and Cerebrospinal Fluid Host Proteins Indicate Stroke in Children With Tuberculous Meningitis. PLoS One (2021) 16(4):e0250944. doi: 10.1371/journal.pone.0250944 33930055 PMC8087017

[207] Schoeman J Mansvelt E Springer P van Rensburg AJ Carlini S Fourie E. Coagulant and Fibrinolytic Status in Tuberculous Meningitis. Pediatr Infect Dis J (2007) 26(5):428–31. doi: 10.1097/01.inf.0000261126.60283.cf 17468654

[208] Jimenez-Alcazar M Rangaswamy C Panda R Bitterling J Simsek YJ Long AT. Host DNases Prevent Vascular Occlusion by Neutrophil Extracellular Traps. Science (2017) 358(6367):1202–6. doi: 10.1126/science.aam8897 29191910

[209] Hakkim A Furnrohr BG Amann K Laube B Abed UA Brinkmann V. Impairment of Neutrophil Extracellular Trap Degradation is Associated With Lupus Nephritis. Proc Natl Acad Sci U S A (2010) 107(21):9813–8. doi: 10.1073/pnas.0909927107 PMC2906830 20439745

[210] Farrera C Fadeel B. Macrophage Clearance of Neutrophil Extracellular Traps is a Silent Process. J Immunol (2013) 191(5):2647–56. doi: 10.4049/jimmunol.1300436 23904163

[211] Brill A Fuchs TA Savchenko AS Thomas GM Martinod K De Meyer SF. Neutrophil Extracellular Traps Promote Deep Vein Thrombosis in Mice. J Thromb Haemost (2012) 10(1):136–44. doi: 10.1111/j.1538-7836.2011.04544.x PMC3319651 22044575

[212] Maugeri N Campana L Gavina M Covino C De Metrio M Panciroli C. Activated Platelets Present High Mobility Group Box 1 to Neutrophils, Inducing Autophagy and Promoting the Extrusion of Neutrophil Extracellular Traps. J Thromb Haemost (2014) 12(12):2074–88. doi: 10.1111/jth.12710 25163512

[213] von Bruhl ML Stark K Steinhart A Chandraratne S Konrad I Lorenz M. Monocytes, Neutrophils, and Platelets Cooperate to Initiate and Propagate Venous Thrombosis in Mice *In Vivo* . J Exp Med (2012) 209(4):819–35. doi: 10.1084/jem.20112322 PMC3328366 22451716

[214] McDonald B Davis RP Kim SJ Tse M Esmon CT Kolaczkowska E. Platelets and Neutrophil Extracellular Traps Collaborate to Promote Intravascular Coagulation During Sepsis in Mice. Blood (2017) 129(10):1357–67. doi: 10.1182/blood-2016-09-741298 PMC5345735 28073784

[215] Laridan E Denorme F Desender L Francois O Andersson T Deckmyn H. Neutrophil Extracellular Traps in Ischemic Stroke Thrombi. Ann Neurol (2017) 82(2):223–32. doi: 10.1002/ana.24993 28696508

[216] Thalin C Hisada Y Lundstrom S Mackman N Wallen H. Neutrophil Extracellular Traps: Villains and Targets in Arterial, Venous, and Cancer-Associated Thrombosis. Arterioscler Thromb Vasc Biol (2019) 39(9):1724–38. doi: 10.1161/ATVBAHA.119.312463 PMC6703916 31315434

[217] Gould TJ Vu TT Swystun LL Dwivedi DJ Mai SH Weitz JI. Neutrophil Extracellular Traps Promote Thrombin Generation Through Platelet-Dependent and Platelet-Independent Mechanisms. Arterioscler Thromb Vasc Biol (2014) 34(9):1977–84. doi: 10.1161/ATVBAHA.114.304114 25012129

[218] Carestia A Kaufman T Schattner M. Platelets: New Bricks in the Building of Neutrophil Extracellular Traps. Front Immunol (2016) 7:271. doi: 10.3389/fimmu.2016.00271 27458459 PMC4933697

[219] Brill A Fuchs TA Chauhan AK Yang JJ De Meyer SF Kollnberger M. Von Willebrand Factor-Mediated Platelet Adhesion is Critical for Deep Vein Thrombosis in Mouse Models. Blood (2011) 117(4):1400–7. doi: 10.1182/blood-2010-05-287623 PMC3056477 20959603

[220] Etulain J Martinod K Wong SL Cifuni SM Schattner M Wagner DD. P-Selectin Promotes Neutrophil Extracellular Trap Formation in Mice. Blood (2015) 126(2):242–6. doi: 10.1182/blood-2015-01-624023 PMC4497964 25979951

[221] Hamzeh-Cognasse H Damien P Chabert A Pozzetto B Cognasse F Garraud O. Platelets and Infections - Complex Interactions With Bacteria. Front Immunol (2015) 6:82. doi: 10.3389/fimmu.2015.00082 25767472 PMC4341565

[222] van der Maten E de Bont CM de Groot R de Jonge MI Langereis JD van der Flier M. Alternative Pathway Regulation by Factor H Modulates Streptococcus Pneumoniae Induced Proinflammatory Cytokine Responses by Decreasing C5a Receptor Crosstalk. Cytokine (2016) 88:281–6. doi: 10.1016/j.cyto.2016.09.025 27721145

[223] Noubouossie DF Whelihan MF Yu YB Sparkenbaugh E Pawlinski R Monroe DM. *In Vitro* Activation of Coagulation by Human Neutrophil DNA and Histone Proteins But Not Neutrophil Extracellular Traps. Blood (2017) 129(8):1021–9. doi: 10.1182/blood-2016-06-722298 PMC5324715 27919911

[224] Semeraro F Ammollo CT Morrissey JH Dale GL Friese P Esmon NL. Extracellular Histones Promote Thrombin Generation Through Platelet-Dependent Mechanisms: Involvement of Platelet TLR2 and TLR4. Blood (2011) 118(7):1952–61. doi: 10.1182/blood-2011-03-343061 PMC3158722 21673343

[225] Kambas K Mitroulis I Apostolidou E Girod A Chrysanthopoulou A Pneumatikos I. Autophagy Mediates the Delivery of Thrombogenic Tissue Factor to Neutrophil Extracellular Traps in Human Sepsis. PLoS One (2012) 7(9):e45427. doi: 10.1371/journal.pone.0045427 23029002 PMC3446899

[226] Massberg S Grahl L von Bruehl ML Manukyan D Pfeiler S Goosmann C. Reciprocal Coupling of Coagulation and Innate Immunity *via* Neutrophil Serine Proteases. Nat Med (2010) 16(8):887–96. doi: 10.1038/nm.2184 20676107

[227] Sambrano GR Huang W Faruqi T Mahrus S Craik C Coughlin SR. Cathepsin G Activates Protease-Activated Receptor-4 in Human Platelets. J Biol Chem (2000) 275(10):6819–23. doi: 10.1074/jbc.275.10.6819 10702240

[228] Shiogama K Onouchi T Mizutani Y Sakurai K Inada K Tsutsumi Y. Visualization of Neutrophil Extracellular Traps and Fibrin Meshwork in Human Fibrinopurulent Inflammatory Lesions: I. Light Microscopic Study. Acta Histochem Cytochem (2016) 49(4):109–16. doi: 10.1267/ahc.16015 PMC5011235 27682014

[229] Onouchi T Shiogama K Mizutani Y Takaki T Tsutsumi Y. Visualization of Neutrophil Extracellular Traps and Fibrin Meshwork in Human Fibrinopurulent Inflammatory Lesions: III. Correlative Light and Electron Microscopic Study. Acta Histochem Cytochem (2016) 49(5):141–7. doi: 10.1267/ahc.16028 PMC5130345 27917008

[230] Onouchi T Shiogama K Matsui T Mizutani Y Sakurai K Inada K. Visualization of Neutrophil Extracellular Traps and Fibrin Meshwork in Human Fibrinopurulent Inflammatory Lesions: II. Ultrastructural Study. Acta Histochem Cytochem (2016) 49(4):117–23. doi: 10.1267/ahc.16016 PMC5011236 27682015

[231] Fuchs TA Brill A Duerschmied D Schatzberg D Monestier M Myers DD Jr. Extracellular DNA Traps Promote Thrombosis. Proc Natl Acad Sci U S A (2010) 107(36):15880–5. doi: 10.1073/pnas.1005743107 PMC2936604 20798043

[232] Thammavongsa V Kim HK Missiakas D Schneewind O. Staphylococcal Manipulation of Host Immune Responses. Nat Rev Microbiol (2015) 13(9):529–43. doi: 10.1038/nrmicro3521 PMC4625792 26272408

[233] Thwaites G Fisher M Hemingway C Scott G Solomon T Innes J. British Infection Society Guidelines for the Diagnosis and Treatment of Tuberculosis of the Central Nervous System in Adults and Children. J Infect (2009) 59(3):167–87. doi: 10.1016/j.jinf.2009.06.011 19643501

[234] van Toorn R Schaaf HS Laubscher JA van Elsland SL Donald PR Schoeman JF. Short Intensified Treatment in Children With Drug-Susceptible Tuberculous Meningitis. Pediatr Infect Dis J (2014) 33(3):248–52. doi: 10.1097/INF.0000000000000065 24168978

[235] Thwaites GE Lan NT Dung NH Quy HT Oanh DT Thoa NT. Effect of Antituberculosis Drug Resistance on Response to Treatment and Outcome in Adults With Tuberculous Meningitis. J Infect Dis (2005) 192(1):79–88. doi: 10.1086/430616 15942897

[236] Donald PR. Cerebrospinal Fluid Concentrations of Antituberculosis Agents in Adults and Children. Tuberculosis (Edinb) (2010) 90(5):279–92. doi: 10.1016/j.tube.2010.07.002 20709598

[237] Ruslami R Ganiem AR Dian S Apriani L Achmad TH van der Ven AJ. Intensified Regimen Containing Rifampicin and Moxifloxacin for Tuberculous Meningitis: An Open-Label, Randomised Controlled Phase 2 Trial. Lancet Infect Dis (2013) 13(1):27–35. doi: 10.1016/S1473-3099(12)70264-5 23103177

[238] Heemskerk AD Bang ND Mai NT Chau TT Phu NH Loc PP. Intensified Antituberculosis Therapy in Adults With Tuberculous Meningitis. N Engl J Med (2016) 374(2):124–34. doi: 10.1056/NEJMoa1507062 26760084

[239] Dian S Yunivita V Ganiem AR Pramaesya T Chaidir L Wahyudi K. Double-Blind, Randomized, Placebo-Controlled Phase II Dose-Finding Study To Evaluate High-Dose Rifampin for Tuberculous Meningitis. Antimicrob Agents Chemother (2018) 62(12). doi: 10.1128/AAC.01014-18 PMC6256787 30224533

[240] Schoeman JF Van Zyl LE Laubscher JA Donald PR. Serial CT Scanning in Childhood Tuberculous Meningitis: Prognostic Features in 198 Cases. J Child Neurol (1995) 10(4):320–9. doi: 10.1177/088307389501000417 7594269

[241] Ramachandran P Duraipandian M Nagarajan M Prabhakar R Ramakrishnan CV Tripathy SP. Three Chemotherapy Studies of Tuberculous Meningitis in Children. Tubercle (1986) 67(1):17–29. doi: 10.1016/0041-3879(86)90028-0 3715980

[242] Alarcon F Escalante L Perez Y Banda H Chacon G Duenas G. Tuberculous Meningitis. Short Course of Chemotherapy. Arch Neurol (1990) 47(12):1313–7. doi: 10.1001/archneur.1990.00530120057010 2252448

[243] Dian S Hermawan R van Laarhoven A Immaculata S Achmad TH Ruslami R. Brain MRI Findings in Relation to Clinical Characteristics and Outcome of Tuberculous Meningitis. PLoS One (2020) 15(11):e0241974. doi: 10.1371/journal.pone.0241974 33186351 PMC7665695

[244] Tsenova L Singhal A. Effects of Host-Directed Therapies on the Pathology of Tuberculosis. J Pathol (2020) 250(5):636–46. doi: 10.1002/path.5407 32108337

[245] Mai NT Dobbs N Phu NH Colas RA Thao LT Thuong NT. A Randomised Double Blind Placebo Controlled Phase 2 Trial of Adjunctive Aspirin for Tuberculous Meningitis in HIV-Uninfected Adults. Elife (2018) 7. doi: 10.7554/eLife.33478 PMC5862527 29482717

[246] Misra UK Kalita J Nair PP. Role of Aspirin in Tuberculous Meningitis: A Randomized Open Label Placebo Controlled Trial. J Neurol Sci (2010) 293(1-2):12–7. doi: 10.1016/j.jns.2010.03.025 20421121

[247] Simmons CP Thwaites GE Quyen NT Chau TT Mai PP Dung NT. The Clinical Benefit of Adjunctive Dexamethasone in Tuberculous Meningitis is Not Associated With Measurable Attenuation of Peripheral or Local Immune Responses. J Immunol (2005) 175(1):579–90. doi: 10.4049/jimmunol.175.1.579 15972695

[248] Misra UK Kalita J Kumar M. Safety and Efficacy of Fludrocortisone in the Treatment of Cerebral Salt Wasting in Patients With Tuberculous Meningitis: A Randomized Clinical Trial. JAMA Neurol (2018) 75(11):1383–91. doi: 10.1001/jamaneurol.2018.2178 PMC6248117 30105362

[249] Cresswell FV Musubire AK Johansson Århem KM. “Chapter 6 - Treatment Guidelines for Tuberculosis and Tuberculous Meningitis”. In: Chin JH-C, editor. Tuberculous Meningitis. San Diego: Academic Press (2020). p. 67–101.

[250] Miow QH Vallejo AF Wang Y Hong JM Bai C Teo FS. Doxycycline Host-Directed Therapy in Human Pulmonary Tuberculosis. J Clin Invest (2021) 131(15). doi: 10.1172/JCI141895 PMC8321570 34128838

[251] Majeed S Radotra BD Sharma S. Adjunctive Role of MMP-9 Inhibition Along With Conventional Anti-Tubercular Drugs Against Experimental Tuberculous Meningitis. Int J Exp Pathol (2016) 97(3):230–7. doi: 10.1111/iep.12191 PMC4960576 27385155

[252] Prasad K Singh MB Ryan H. Corticosteroids for Managing Tuberculous Meningitis. Cochrane Database Syst Rev (2016) 4:CD002244. doi: 10.1002/14651858.CD002244.pub4 27121755 PMC4916936

[253] Thwaites GE Macmullen-Price J Tran TH Pham PM Nguyen TD Simmons CP. Serial MRI to Determine the Effect of Dexamethasone on the Cerebral Pathology of Tuberculous Meningitis: An Observational Study. Lancet Neurol (2007) 6(3):230–6. doi: 10.1016/S1474-4422(07)70034-0 PMC4333204 17303529

[254] Torok ME. Tuberculous Meningitis: Advances in Diagnosis and Treatment. Br Med Bull (2015) 113(1):117–31. doi: 10.1093/bmb/ldv003 25693576

[255] Suarez I Gruell H Heyckendorf J Funger S Lichtenstein T Jung N. Intensified Adjunctive Corticosteroid Therapy for CNS Tuberculomas. Infection (2020) 48(2):289–93. doi: 10.1007/s15010-019-01378-3 31900872

[256] Afghani B Lieberman JM. Paradoxical Enlargement or Development of Intracranial Tuberculomas During Therapy: Case Report and Review. Clin Infect Dis (1994) 19(6):1092–9. doi: 10.1093/clinids/19.6.1092 7888539

[257] Anuradha HK Garg RK Sinha MK Agarwal A Verma R Singh MK. Intracranial Tuberculomas in Patients With Tuberculous Meningitis: Predictors and Prognostic Significance. Int J Tuberc Lung Dis (2011) 15(2):234–9. 21219687

[258] Mazodier K Bernit E Faure V Rovery C Gayet S Seux V. [Central Nervous Tuberculosis in Patients non-VIH: Seven Case Reports]. Rev Med Interne (2003) 24(2):78–85. doi: 10.1016/S0248-8663(02)00715-4 12650889

[259] Schoeman JF Janse van Rensburg A Laubscher JA Springer P. The Role of Aspirin in Childhood Tuberculous Meningitis. J Child Neurol (2011) 26(8):956–62. doi: 10.1177/0883073811398132 21628697

[260] Richman IB Owens DK. Aspirin for Primary Prevention. Med Clin North Am (2017) 101(4):713–24. doi: 10.1016/j.mcna.2017.03.004 28577622

[261] Botting RM. Vane's Discovery of the Mechanism of Action of Aspirin Changed Our Understanding of its Clinical Pharmacology. Pharmacol Rep (2010) 62(3):518–25. doi: 10.1016/S1734-1140(10)70308-X 20631416

[262] Raju N Sobieraj-Teague M Hirsh J O'Donnell M Eikelboom J. Effect of Aspirin on Mortality in the Primary Prevention of Cardiovascular Disease. Am J Med (2011) 124(7):621–9. doi: 10.1016/j.amjmed.2011.01.018 21592450

[263] Ortiz-Munoz G Mallavia B Bins A Headley M Krummel MF Looney MR. Aspirin-Triggered 15-Epi-Lipoxin A4 Regulates Neutrophil-Platelet Aggregation and Attenuates Acute Lung Injury in Mice. Blood (2014) 124(17):2625–34. doi: 10.1182/blood-2014-03-562876 PMC4208278 25143486

[264] Kroesen VM Rodriguez-Martinez P Garcia E Rosales Y Diaz J Martin-Cespedes M. A Beneficial Effect of Low-Dose Aspirin in a Murine Model of Active Tuberculosis. Front Immunol (2018) 9:798. doi: 10.3389/fimmu.2018.00798 29740435 PMC5924809

[265] Lapponi MJ Carestia A Landoni VI Rivadeneyra L Etulain J Negrotto S. Regulation of Neutrophil Extracellular Trap Formation by Anti-Inflammatory Drugs. J Pharmacol Exp Ther (2013) 345(3):430–7. doi: 10.1124/jpet.112.202879 23536315

[266] Hamid U Krasnodembskaya A Fitzgerald M Shyamsundar M Kissenpfennig A Scott C. Aspirin Reduces Lipopolysaccharide-Induced Pulmonary Inflammation in Human Models of ARDS. Thorax (2017) 72(11):971–80. doi: 10.1136/thoraxjnl-2016-208571 PMC5858553 28082531

